# Multivalent Interactions between Chaperone and Ribosome-Nascent Chain Complex Revealed by High-Speed AFM and MD Simulations

**DOI:** 10.1021/acsnano.5c13500

**Published:** 2025-12-11

**Authors:** Eider Nuñez, Prithwidip Saha, Markel G. Ibarluzea, Arantza Muguruza-Montero, Sara M-Alicante, Rafael Ramis, Aritz Leonardo, Aitor Bergara, Alvaro Villarroel, Felix Rico

**Affiliations:** † Aix-Marseille University, INSERM, DyNaMo, Turing Centre For Living Systems, Marseille 13009, France; ‡ Biofisika Institute, CSIC-UPV/EHU, Leioa 48940, Spain; § 226245 Donostia International Physics Center, Donostia 20018, Spain; ∥ Department of Physics and EHU Quantum Center, University of The Basque Country, UPV/EHU, Leioa 48940, Spain; ⊥ Department of Physiology, Faculty of Medicine and Nursery, University of the Basque Country, UPV/EHU, Leioa 48940, Spain; # Materials Physics Center, 20018 Donostia, Spain

**Keywords:** atomic force microscopy imaging, molecular dynamics simulations, ribosome, trigger factor (chaperone), protein folding

## Abstract

Trigger Factor (TF) is a primary ATP-independent molecular chaperone in bacteria that engages nascent polypeptide chains emerging from the ribosomal exit tunnel to assist their folding. However, the real-time behavior of TF during active translation under near-physiological conditions remains elusive. Here, we employ high-speed atomic force microscopy (HS-AFM) imaging to visualize TF dynamics on intact *Escherichia coli* ribosomes in real time. We observe that TF transitions between compact and extended conformations and forms stable and transient contacts near ribosomal proteins uL23 and bL17, respectively. Interestingly, TFs engage distinct regions of the same ribosome–nascent chain complex, with one TF binding near the nascent chain and another near bL17, revealing multivalent interactions on the ribosome surface. Complementary all-atom molecular dynamics simulations reproduced the observed TF conformations and interaction dynamics, validating the experimentally observed structural transitions and dual-site engagement. This integrative approach uncovers previously inaccessible dynamics of ribosome-associated chaperones and offers a broadly applicable platform to probe cotranslational folding under near-physiological conditions.

## Graphical abstract

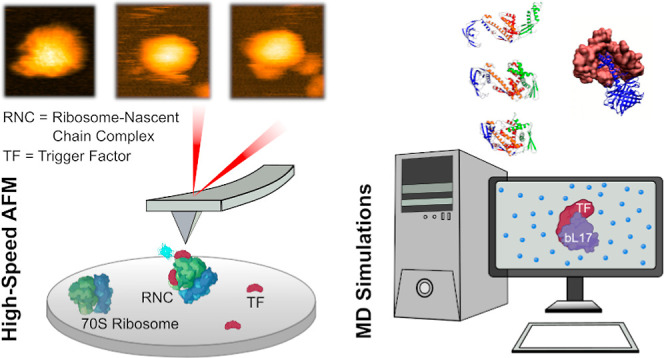

## Introduction

Protein synthesis is a central process in which ribosomes decode mRNA into polypeptides that fold into functional structures. In prokaryotes, the ribosome comprises two subunits (30S and 50S) that coordinate mRNA and tRNAs binding during translation. Structurally, the ribosome features distinct domains that contribute to its function, including the L1 stalk, involved in tRNA release;^1^ the protein stalk or P-stalk (Ps), which recruits translation factors;^2^ the central protuberance (CP), a scaffold for tRNA positioning;^3^ and the 30S spur (sp), a flexible extension implicated in ribosomal activity.^4^ As the nascent chain (NC) emerges through the ribosomal exit tunnel, protein misfolding may occur.^5^ To prevent this, cells rely on molecular chaperones that assist cotranslational folding, preserving proteostasis and ensuring functional protein output.^6–8^

Trigger Factor (TF) is the main ATP-independent, ribosome-associated chaperone in bacteria, binding near the exit tunnel to prevent NC aggregation and assist folding.^9–13^ It contains an N-terminal ribosome-binding domain (RBD), a central PPIase domain, and a C-terminal substrate-binding domain (SBD) with two flexible “arms” that cradle the emerging polypeptide;^14,15,16,17,18^ both the RBD and SBD are essential for its activity.^19–21^ Biochemical and structural studies have established that TF binds near ribosomal protein uL23 at the tunnel exit, providing a key docking site for cotranslational chaperone function.^14,15^ High-resolution static structures of ribosome–chaperone complexes provide invaluable insights into the molecular architecture of these interactions but inherently fail to capture the dynamic, real-time behavior of chaperones during active translation.^15,17,21,22^ Complementary approaches, including X-ray crystallography, NMR spectroscopy, and cryo-EM, have highlighted the pronounced conformational flexibility of the TF, showing that it adapts to both ribosomal functional states and nascent chain properties.^15–17^ However, these approaches do not directly visualize TF dynamics, leaving key questions about how TF associates, dissociates, undergoes conformational transitions, and selectively engages with translating ribosomes still open. This gap limits our understanding of the exact molecular mechanisms by which TF recognizes nascent polypeptides and ensures their proper cotranslational folding.

High-speed atomic force microscopy (HS-AFM) has emerged as a powerful tool to image macromolecular complexes in solution with nanometer spatial and subsecond temporal resolution under near-physiological conditions.^23–27^ A pioneering study by Imai et al. reported real-time visualization of an engineered archaeal 50S subunit and GTPase binding to its P-stalk,^27,28^ confirming that HS-AFM offers an unprecedented window into the dynamics of bio macromolecular complexes in a native-like environment. Nevertheless, direct visualization of intact, translating *Escherichia coli* ribosomes remains a challenge, yet it would uniquely bridge existing static structural models and dynamic functional insights into ribosome–TF interactions in situ.

Here, we used HS-AFM to directly visualize TF dynamics on ribosome-nascent chain complexes (RNCs). By combining HS-AFM with all-atom molecular dynamics (MD) simulations, we captured TF’s real-time conformational transitions and observed stable and transient interactions near ribosomal proteins uL23 and bL17, respectively, on translating *E. coli* ribosomes. Our approach enables direct localization and dynamic characterization of TF and 70S ribosomes in a native context, without engineered constructs, establishing a new framework for probing translation-associated factors with nanometer resolution.

## Results and Discussion

### HS-AFM Imaging of Intact 70S Ribosomes Reveals Structural Features on Different Orientations

We isolated native 70S ribosomes from *E. coli* using an optimized two-step protocol (see Materials and Methods). To preserve ribosomal integrity during HS-AFM imaging, we optimized scanning conditions, enabling high-resolution visualization of intact ribosomes. An imaging buffer containing MgAc provided sufficient stabilization to prevent subunit dissociation while maintaining native conformations.^29–32^ Bare mica, as a surface, offered effective electrostatic interaction with the MgAc-stabilized ribosomes, allowing partial immobilization and real-time imaging of their structural dynamics under near-physiological conditions (Figure 1 and Videos S1–S5). This approach yielded multiple ribosome orientations on the surface, a key outcome of the native preparation that facilitated comprehensive structural analysis and allowed the direct visualization of ribosome dynamics, capturing the complex in a near-functional state.

**Figure 1:**
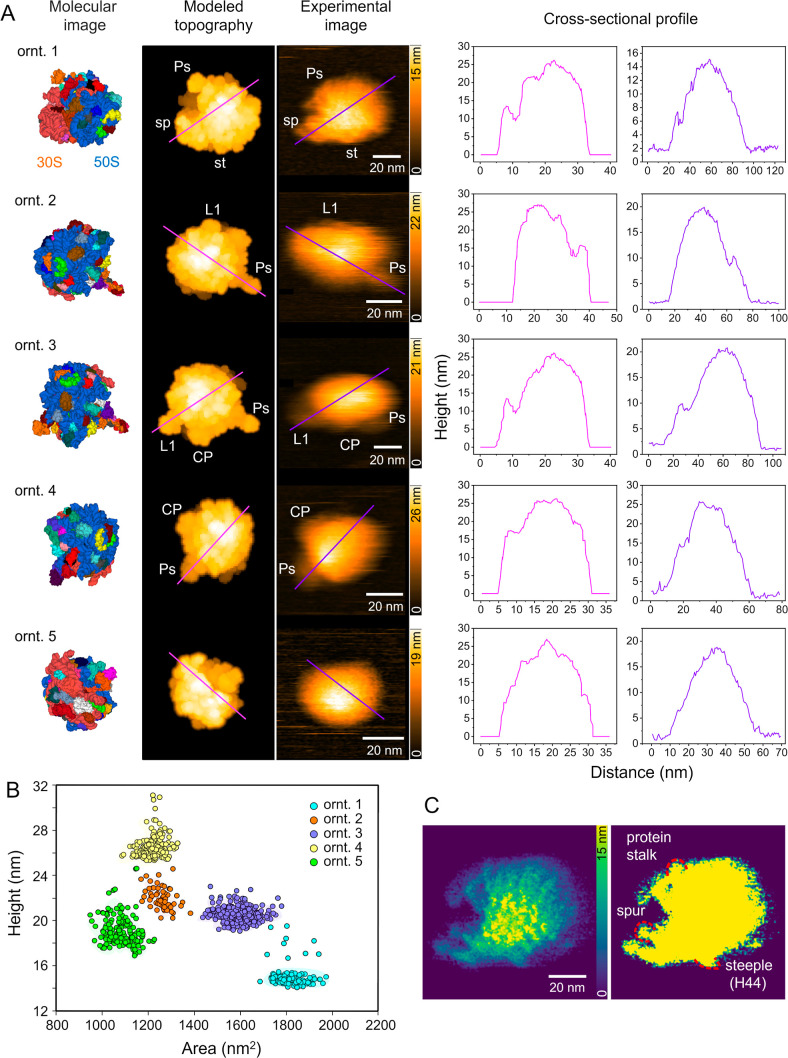
Visualization of the 70S ribosome in various orientations on a mica surface. (A) Experimental HS-AFM images (third panel) of the 70S ribosome captured in five different orientations. Molecular images (first panel) and modeled topographies (second panel) were generated from the structure of the translating bacterial ribosome (PDB: 7K00),^35^ using a probe tip radius of 1 nm and a cone angle of 5°. In the molecular image, the 50S subunit was colored blue and the 30S subunit coral. In the second and third panels, key structural features were labeled: sp = spur, L1 stalk, CP = central protuberance, Ps = protein stalk, and st = steeple. The cross-sectional profiles of modeled topography (magenta) and experimental image (purple) for the five orientations are shown in the fourth panel. (B) A plot showing the 70S ribosome’s height and projected area measurements. Each data point represented a single ribosome that was assigned to one of the five orientations shown in (A). See Figure S1A for the distribution of the entire population obtained during AFM experiments. (C) A high-resolution map of ribosome orientation 1 was generated by applying the LAFM image processing algorithm across multiple consecutive frames.^38,39^ Distinct structural features, including the spur, the protein stalk, and the steeple (H44), are highlighted with red dashed lines. (left) Gamma correction was applied to enhance the image intensity; (right) contrast was adjusted to improve the clarity of features around the ribosomal peripheral regions.

To interpret ribosomal topographical features, we used BioAFMviewer^33,34^ to generate modeled AFM images from ribosomal molecular structure and to perform rigid-body fitting that closely aligned the molecular structure with our experimental HS-AFM images. For that, we used the high-resolution structure of the 70S ribosome (PDB: 7K00 ^35^), complemented with the P-stalk (PDB: 1ZAW^36^) as the molecular structure. The comparison between the resulting modeled AFM images with the experimental data, along with the analysis of cross-sectional profiles from both image types (Figure 1A), allowed us to reliably identify key structural features, including the 30S sp, Ps, and 50S CP (Figure 1A). As expected for the modeled AFM data, the apparent height values occasionally exceeded the experimental values due to the rigid modeling of deformable structures, an effect that may also account for the larger width observed in experimental ribosome cross-sectional profiles (Figure 1A).^34^ Nevertheless, the strong structural correlation between the modeled and the experimental AFM topographies supports our orientation assignment. The corresponding measurements of height and projected area further validated these assignments (Figure 1B).

In orientation 1 (Figure 1A), both the 50S and 30S subunits of the ribosome lay side by side facing the probe tip, resulting in a topographical height profile dominated by the 50S subunit but a total volume consistent with the intact 70S complex (Figures 1B and S1A). This orientation also exposed the 30S head and revealed flexibility in peripheral regions, such as the spur, corresponding to helix 6 of the 16S rRNA (Figure S1B). The dynamic behaviors of the spur, until now, had been accessible only via computational inference.^37^

To enhance the spatial resolution of HS-AFM topography images, we applied a localization AFM (LAFM) reconstruction algorithm across multiple AFM frames, resulting in a LAFM map with improved molecular detail (Figure 1C).^38,39^ This approach extends the lateral resolution of our AFM topographs to the scale of individual protein- and RNA-rich regions. For example, we observed a small, recurring protrusion at the intersubunit interface in the LAFM map (Figure 1C), which we ascribed to be the h44 helix, also referred to as the “steeple” due to its protruding shape.^40^ However, the exact correspondence with the Cryo-EM structures remained difficult.

The ribosomal orientations 2 and 3 revealed topographical features consistent with the CP and Ps region. The Ps exhibited considerable flexibility (Figure S1C), in agreement with previous reports on ribosomal dynamics^2,27^ (Figure 1, Videos S2 and S3). This dynamic behavior was further exemplified in orientation 4, which revealed pronounced motion of the Ps (Figure 1, Video S4). The observed angular movement (∼90°–140° in our native *E. coli* 70S ribosomes, Figure S1C) was consistent with, though narrower than, the range reported by Imai and co-workers for an engineered archaeal 50S subunit (∼80°–240°).^27^ This difference likely stems from steric constraints imposed by the presence of the 30S subunit in our intact 70S complexes, although variations in buffer composition or inherent structural differences between the archaeal hybrid and native bacterial stalks may also contribute. Notably, the L1 stalk was clearly discernible in the third orientation, underscoring the capability of HS-AFM to resolve dynamic peripheral ribosomal features (Video S3). Finally, we identified a fifth recurring orientation characterized by a globular appearance without clearly discernible peripheral features (Figure 1). The lack of distinguishable topographical features in this orientation makes it difficult to definitely correspond with the 70S ribosome molecular structure. Therefore, we classified this orientation as a distinct morphological state representative of the ribosome’s architecture in general, which was mostly observed on the surface.

The 70S ribosome exhibited an average height of 21 ± 4 nm, while the isolated 50S subunit measured 15 ± 1 nm (Figure S1A), in agreement with previous reports of ribosomes imaged in solution^27,41^ and in air.^42^ The average projected area was 1552 ± 238 nm^2^ for the 70S ribosome and 946 ± 169 nm^2^ for the 50S subunit (Figure S1A).

Although ribosomes are often described as roughly spherical at low resolution, their 3D architecture is intrinsically asymmetric, as previously shown.^35^ This anisotropy results in orientation-dependent variations in height and projected area in HS-AFM images, consistent with prior analyses of individual subunits and intact ribosomes.^27,41,42^ In our data set, 70S ribosomes exhibited distinct height–area profiles across 5 different orientations on the mica surface. Notably, ribosomes exhibiting greater heights consistently exhibited smaller projected areas, a reflection of their inherently nonspherical geometry.

To estimate the volume (*V*) of individual complexes more accurately, we applied a spherical cap approximation, using the expression

$$V=\frac{\pi h}{6}\left(\frac{3A}{\pi }+h^{2}\right)$$

where, *h* is the measured height of the complex and *A* the projected area derived from HS-AFM images. This approach yielded orientation-specific mean volumes of 15.5 ± 1.5, 19.72 ± 3.3, 21.5 ± 1.3, 25.6 ± 1.8, and 16.2 ± 3.0 × 10^3^ nm^3^ for orientations 1 to 5 (*n* = 201, 64, 199, 199, 209, respectively). As independent cross-validation, we performed simulated AFM scanning of the PDB: 7K00 (including its P-stalk) in BioAFMviewer, and it provided slightly lower volumes: 12.9, 16.7, 17.3, 20.0, and 15.2 × 10^3^ nm^3^ for the same orientations. We attribute this difference as an overestimation in the experimental AFM imaging due to tip convolution effects, where the finite size of the AFM tip broadens the lateral dimensions.^43^ Our analysis (Figure S1D) confirms that a tip radius of 3–4 nm, consistent with the etched ultrashort cantilevers used in our case, yields simulated volumes in close agreement with our experimental measurements. The agreement between both approaches supports the interpretation that these biomolecular complexes are intact 70S ribosomes. In contrast, dissociated 50S subunits (*n* = 935) displayed significantly lower volumes (9.2 ± 2.6 × 10^3^ nm^3^) (Figure S1A).

### Visualization of Conformational Dynamics of TF Using MD and HS-AFM

To characterize the intrinsic conformational flexibility of the TF monomer, we combined all-atom MD simulations with HS-AFM imaging. Simulations were initiated from the crystal structure of TF reflecting an extended conformation (PDB: 1T11 ^44^) and comprised four independent MD runs using two force-field/solvent combinations (ff19SB/OPC^45,46^ and CHARMM36/TIP3P;^47,48^ see Materials and Methods). Analysis of solvent-accessible surface area (SASA) traces reveals large-scale conformational excursions of TF over the simulated interval (Figure 2A, top; Videos S6 and S7). Representative snapshots extracted from a CHARMM36 trajectory illustrate a clear progression from an extended architecture (frames 1–3), through a semicompact intermediate (frames 4–6), to a fully compact arrangement (frames 7–10) (Figure 2A, bottom). To quantitatively define the conformational states sampled during MD simulations, we analyzed the SASA evolution from our 3 μs CHARMM36 trajectory (53,000 frames). Histogram analysis of SASA values revealed a trimodal distribution that we decomposed into three Gaussian components, each corresponding to a distinct conformational state (Figure S2A). The intersection points between adjacent Gaussians provided thresholds for conformational state classification: Compact (C): SASA <273 nm^2^ (∼83.2%), Semicompact (SC): 273 nm^2^ ≤ SASA ≤286 nm^2^ (∼13.8%), and Extended (E): SASA >286 nm^2^ (∼3%). Inspection of interdomain contacts shows that the early semicompaction is marked by transient, reversible contacts between the PPIase domain and C-terminal arm 1, whereas the compact state is distinguished by a more persistent interface formed between the N-terminal domain and both the C-terminal arms; in several trajectories one C-terminal arm adopts a layered, sandwiched geometry. These observations were consistent across both force-field/solvent combinations, indicating robustness to the simulation model and are in line with the conformational flexibility reported for TF.^49–51^

**Figure 2:**
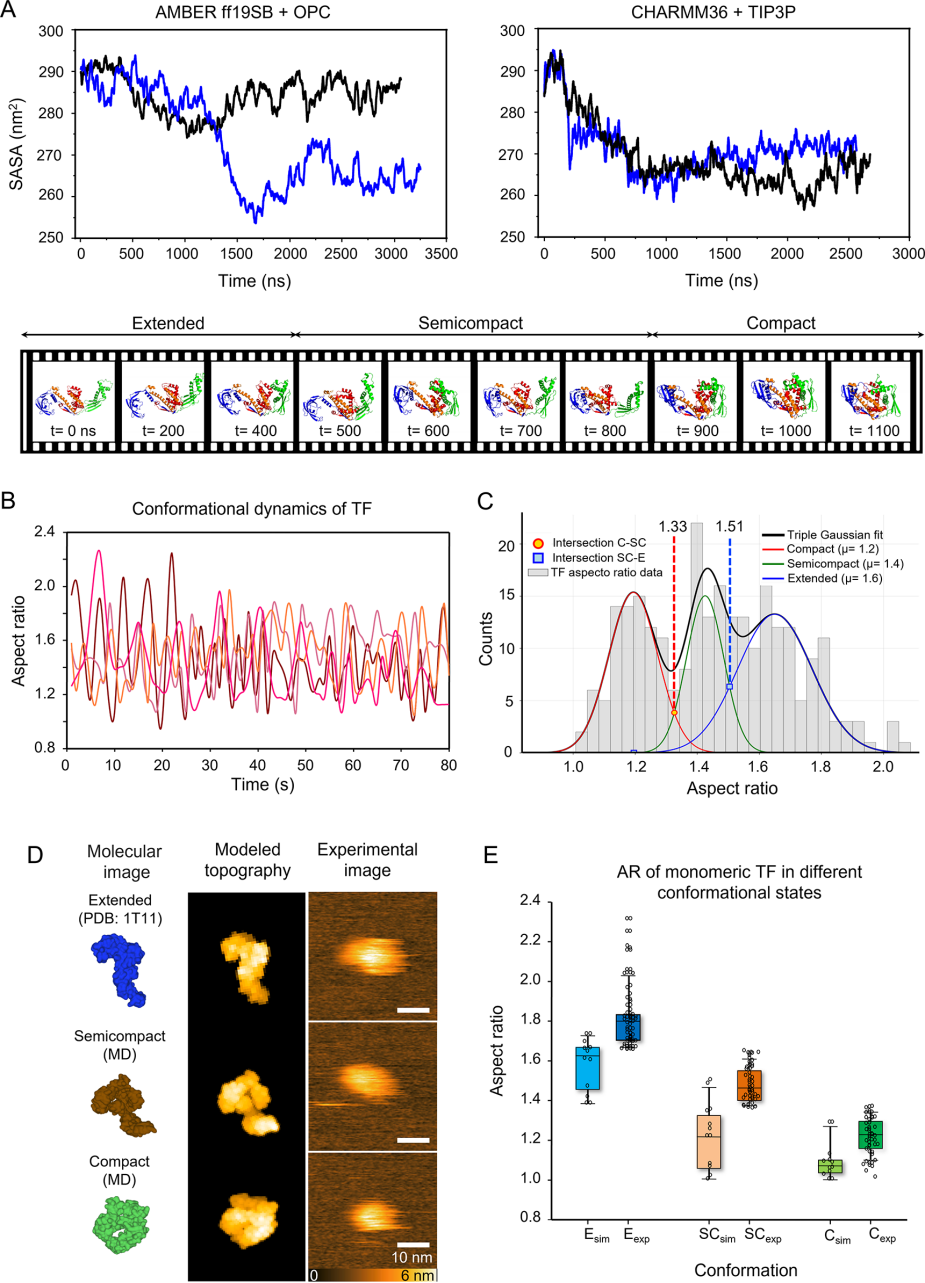
Conformational flexibility of the TF monomer in solution. (A) Top: solvent accessible surface area (SASA) plots of the TF monomer over time from four independent simulations, blue and black lines represent different simulation replicates within each force field. The left panel presents results obtained using the AMBERrff19SB + OPC force field, while the right panel shows results from the CHARMM36 + TIP3P force field. Bottom: sequential MD snapshots showing conformational transitions of the TF monomer. Ten representative frames extracted from a single MD trajectory are shown in chronological order. It captures the transition of TF from the extended (E) conformation (frames 1–3), through a semicompact (SC) intermediate state (frames 4–6), to the fully compact (C) conformation (frames 7–10). TF domains are color-coded: N-terminal domain (green), PPIase domain (blue), and C-terminal arms 1 and 2 (orange and red, respectively). During the SC state, the first key event involves transient, reversible contacts between the PPIase domain and C-terminal arm 1, marking the onset of compaction. In the C state, a second, more stable event is observed: formation of a persistent interface between the N-terminal domain and both C-terminal arms. In some cases, one C-terminal arm becomes sandwiched in a layered arrangement. Occasional persistent contacts involving the PPIase domain may also follow. (B) Time-dependent variation in the aspect ratio (AR) of the TF monomer at a concentration of 5 nM in physiological buffer (25 mM Tris, pH 7.5, 120 mM KCl, 5 mM NaCl, and 14 mM MgAc). Data from different experiments are represented in different shades of red. (C) Histogram of TF conformational states based on the experimentally measured AR. The distribution was fitted using a Gaussian mixture model (*R* ^2^ = 0.82), revealing three distinct populations: compact (AR < 1.33; ∼32.8%), semicompact (1.33≤ AR ≤ 1.50; ∼24.2%), and extended (AR > 1.50; ∼43.0%). Vertical dashed lines indicate the threshold values at the intersections of the fitted Gaussian curves (∼1.33, marked with red and ∼1.50, marked with blue). (D) Molecular representations of three distinct TF conformations (first panel): extended (top, initial state, PDB: 1T11, only a monomeric chain was used), semicompact (middle, at 490 ns of CHARMM36 simulation), and compact (bottom, 2100 ns of CHARMM36 simulation). Modeled topographies (second panel) were generated with BioAFMviewer, using a probe tip radius of 1 nm and a cone angle of 5°. Experimental HS-AFM images are shown in the third panel. (E) Boxplots of MD simulation and experimental AR measurements for the extended (E, blue), semicompact (SC, green), and compact (C, red) conformations. The black lines represent the standard deviation. Experimental ARs (exp) are represented in solid colors, while simulated ARs (sim) are shown in lighter shades, with individual data points representing one of these three conformations obtained from the different AFM frames and MD simulations. AR values from simulated molecules were calculated using BioAFMviewer and analyzed across at least 10 different orientations, obtained from the CHARMM36 + TIP3P trajectory at *t* = 0 ns (extended), *t* = 490 ns (semicompact), and *t* = 2100 ns (compact). Experimental ARs were determined by dividing FeretY by FeretX, with classification thresholds of >1.5 for the extended state, 1.5–1.33 for the semicompact state, and <1.33 for the compact state.

To relate the MD-derived states to experimentally accessible observables, we imaged TF monomers by HS-AFM (5 nM) in physiological buffer (25 mM Tris, pH 7.5; 120 mM KCl; 5 mM NaCl; and 14 mM MgAc) (Video S8). Representative single-molecule time traces of the aspect ratio (AR = FeretY/FeretX) show dynamic interconversion between more elongated and more compact appearances on the seconds time scale (Figure 2B). Traces from independent experiments indicate that individual molecules sample a broad range of AR values during the observation time window. We quantified the AR from a large set of HS-AFM frames and fitted the histogram with a three-component Gaussian Mixture Model (Figure 2C). The decomposition yields three distinct populations, which we classified as compact (μ = 1.19), semicompact (μ = 1.43), and extended (μ = 1.65). The intersections between the fitted Gaussians provided objective thresholds for state separation at AR values of ∼ 1.33 and ∼ 1.50 (R^2^ = 0.82), which were used for frame-by-frame assignment. Based on the area under each curve, the relative populations are classified as compact (AR < 1.33; ∼32.8%), semicompact (1.33≤ AR ≤ 1.50; ∼24.2%), and extended (AR > 1.50; ∼43.0%). The predominance of extended and compact conformations, alongside a significant population of the semicompact state, defines a conformational landscape for TF that aligns with and refines the multistate models proposed for other chaperone systems.^52–54^

For a direct comparison between experimental and computational data, we extracted representative conformations from the CHARMM36 trajectory (SASA = 293 nm^2^, *t* = 60 ns for E; SASA = 278 nm^2^, *t* = 450 ns for SC; and SASA = 257 nm^2^, *t* = 2200 ns for C) and generated modeled AFM topographies with BioAFMviewer (Figure 2D). We then computed AR values from the modeled topographies over ≥10 orientations per state and compared them with the AR measurements from the HS-AFM images. The resulting boxplots (Figure 2E) show that the simulation-derived and experiment-derived AR distributions occupy overlapping ranges with comparable medians and variability for the three states, supporting the assignment of the experimentally observed populations to the MD-derived extended, semicompact, and compact conformations. Simulation ARs were computed from BioAFMviewer outputs, and experimental ARs were calculated as FeretY/FeretX; classification of experimental frames used the thresholds defined in Figure 2C. Taken together, these data show quantitative agreement between MD and HS-AFM at the level of the aspect ratio metric and support a model in which TF samples have three principal conformational basins. The observed differences in state populations between MD (predominantly compact) and HS-AFM (balanced distribution) likely reflect the distinct time scales and conditions of each technique.

To assess the differences between monomeric and dimeric forms of TF, we promoted dimer formation by increasing the TF concentration to 5 μM. TF self-associates to form a dimer with an equilibrium dissociation constant (*K*_d_) typically found to be 1 to 2 μM^14,55–57^ (Video S9). Under these conditions, dimers displayed substantially larger projected areas (241 ± 89 nm^2^) and greater heights (8.0 ± 1.0 nm) than monomers, which measured 110 ± 17 nm^2^ in the projected area and 6.0 ± 1.0 nm in height (Figure S2A). This increase likely reflects added mass and conformational rearrangement upon dimerization, though HS-AFM alone cannot resolve specific domain interfaces. Notably, the wide distribution of projected areas suggests a dynamic equilibrium in which, alongside dimers, higher-order oligomers may form, as previously described for TF from *Thermotoga maritima* ^58^ (Figure S2A).

The dimer’s AR stayed relatively constant over time (1.3–1.5; Figure S2B, Video S9) and was more uniform than that of the monomer (1.1–2.2; Figure 2A). These findings indicate that TF dimers adopt a structurally stable assembly with reduced flexibility, which is consistent with prior work showing that head-to-tail dimerization preorganizes the substrate-binding cavity and limits nonspecific interactions.^57^ At a high concentration of TF (5 μM), once dimers were formed, they remained stable and did not revert to monomers. This observation is consistent with previous reports showing that, in the absence of targets and ribosomes, TF dimers represent the dominant resting-state populations.^59–61^

### Interaction of TF with Ribosomes

To understand how TF engages translating ribosomes, we first tested its binding capacity in the absence of a nascent polypeptide chain (NC). Under these conditions, TF exhibited minimal association (<5 s), even at a high molar ratio of 200:1 TF:ribosome (Figure S3A, Video S10). Such short-lived contacts have been previously reported in the absence of NCs.^21,22,44,62,63^ We observed stable TF association (>5 s) only in the presence of ribosome–nascent chain complexes (RNCs). This supports a model of substrate-driven TF recruitment, consistent with TF’s affinity for emerging hydrophobic segments,^56,57,64–66^ and highlights the importance of cotranslational context for biologically relevant TF engagement.

To mimic this context, we designed a construct containing an SecM arrest motif (a bacterial peptide that transiently stalls ribosomes to regulate protein secretion) fused to two α-helices and monomeric Teal Fluorescent Protein 1 (mTFP1) (see Materials and Methods and Figure S3B). This configuration stalls translation at a defined site, exposing a nascent chain outside the ribosomal tunnel, where the TF was expected to bind (Figure 3A). The resulting complex produced a distinct topographical feature in high-resolution AFM images and allowed us to identify the translating ribosomes.

**Figure 3:**
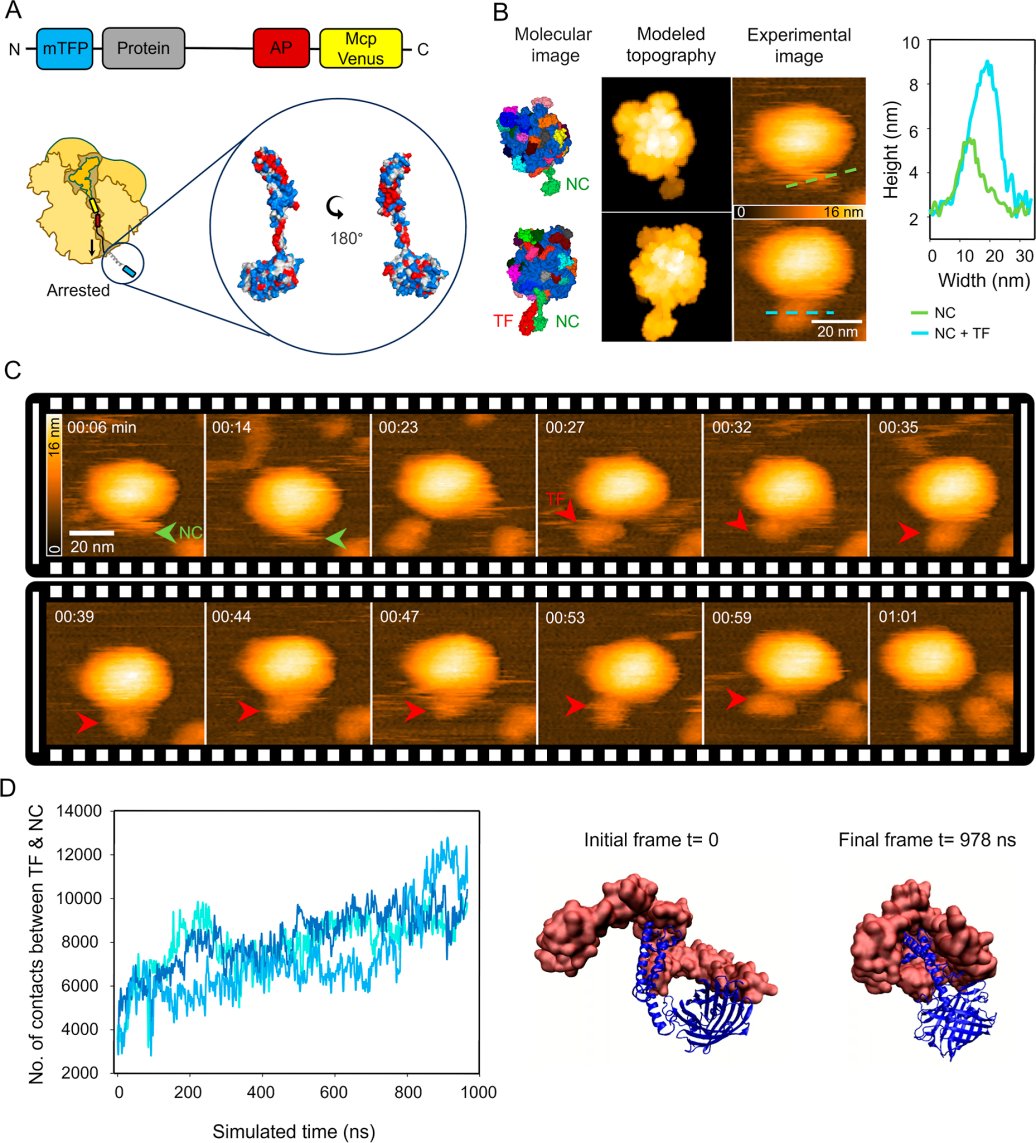
TF binding to a ribosome-nascent chain complex. (A) Schematic representation of the nascent chain used in the TF binding assay. Top: a construct of the nascent chain that included a mTFP fluorescent protein (blue), α-helices with hydrophobic patches (gray), SecM arrest peptide (AP, red), and a nontranslated mcpVenus protein (yellow). Bottom: a ribosome–nascent chain complex (RNC) was shown, with a zoom-in of the AlphaFold2-predicted structure of the accessible portion of the nascent chain. The predicted structure was colored by residue hydrophobicity: red for hydrophobic, blue for hydrophilic, and white for neutral residues. (B) HS-AFM images of RNC with and without TF binding (third panel), along with molecular images (first panel) and modeled topographies (second panel). Modeled AFM topographies from BioAFMviewer (generated using a probe tip radius of 1 nm and a cone angle of 5°) were shown for comparison with the experimental data. On the right, cross-sectional profiles showed the topographic height of the RNC alone (green line) and the TF-bound RNC (cyan line), highlighting the additional volume attributed to TF binding. (C) Sequential frames illustrated a representative TF binding event on an RNC in a physiological buffer (RNC at 2 nM, TF at 1 μM). TF was marked with red arrowheads and NC with green arrowheads. The time stamp was shown at the upper-left corner of each frame. (D) Left: MD simulations depicted the TF–NC interaction. The number of contacts between the NC and TF was tracked using a heavy-atom distance cutoff of 3.0 Å. Traces showed the mean number of contacts over three independent replicas (indicated with different shades of blue), each run for 1000 ns. Right: the initial and final frames of the interaction between the RNC (blue) and TF (coral) revealed how TF embraced the nascent chain.

AlphaFold2 ^67^ modeling of the RNC predicted that the stalled polypeptide includes mTFP1 and helical segments enriched in hydrophobic residues (Figure 3A). Morphometric analysis of the experimentally observed RNCs, which predominantly adopted the fifth orientation (Figure 1A), revealed a height of 17 ± 3 nm. The measured height of the NC protrusion was ∼5 nm, in agreement with the modeled structure (Figure 3B). Upon TF binding, the cross-sectional height of NC increased significantly to 9.6 ± 1.2 nm, accompanied by a lateral broadening from 11 to 20 nm. Biochemical validation of TF and RNC purity (Figure S3B) confirmed the sample integrity prior to AFM imaging. The observed increase in NC height upon TF binding, along with the greater structural flexibility of monomeric TF compared to the more rigid dimer, supports the interpretation that TF engages the NC in its monomeric form. This is consistent with prior reports describing monomeric TF as the active, substrate-binding species, while dimerization has been associated with reduced flexibility and limited nonspecific interactions.^44,55,64–66^ TF binding to the RNC was reasonably stable, with individual binding events lasting over 30 s (Figure 3C, Video S11).

To investigate TF–NC interactions at the molecular level, we conducted multiple 1 μs all-atom MD simulations^68^ using an AlphaFold2-predicted model as the starting structure (Figure 3D, Video S12). Despite the model exhibiting a low confidence score (ipTM + pTM ≈0.19), likely reflecting the intrinsic flexibility of nascent chains and the lack of resolved structural data for this complex, it provided a suitable starting point to investigate general binding modes and dynamics. To minimize bias from the initial model, multiple independent replicas were simulated. The simulations revealed extensive and dynamic contacts with TF primarily engaging hydrophobic regions of the NC. TF adopted a flexible and adaptive configuration, wrapping around the NC in a manner consistent with its conformational versatility observed in previous NMR^66^ and MD^69–72^ studies. The C-terminal domain of TF (residues L248 and I432) played a dominant role in binding, forming persistent, close-range contacts with the alpha helices of the NC.^14,15^ Distance histograms showed a sharp peak at 2.6 Å (Figure S4A), corresponding to the proximity between TF residues 300–392 and the NC. Residues 320 and 377 emerged as key contact points (Figure S4B), placing the interaction within or near arm 2 of the C-terminal domain, which has been implicated in nascent chain recognition.^17,73^ A progressive increase in hydrogen bonding throughout the simulations (Figure S4C) reflects the stabilization of the TF–NC interface in silico. This observation complements the AFM observations of global TF wrapping around the NC, without implying any direct correspondence between the hydrogen-bond number and AFM results.

### Stable and Transient Binding Patterns of TF to RNC

Using HS-AFM, we identified binding of two TF molecules to the same RNC (Figure 4A–C, Figure S5), revealing a more complex stoichiometry than the canonical 1:1 TF–ribosome model.^10,11,21,62,74^ Across multiple ribosome orientations, one TF consistently anchors at a site we identified as the exit-tunnel vestibule (see MD simulations below), while another transiently interacts with a second site on the peripheral ribosomal surface (Figure 4A,B).

**Figure 4:**
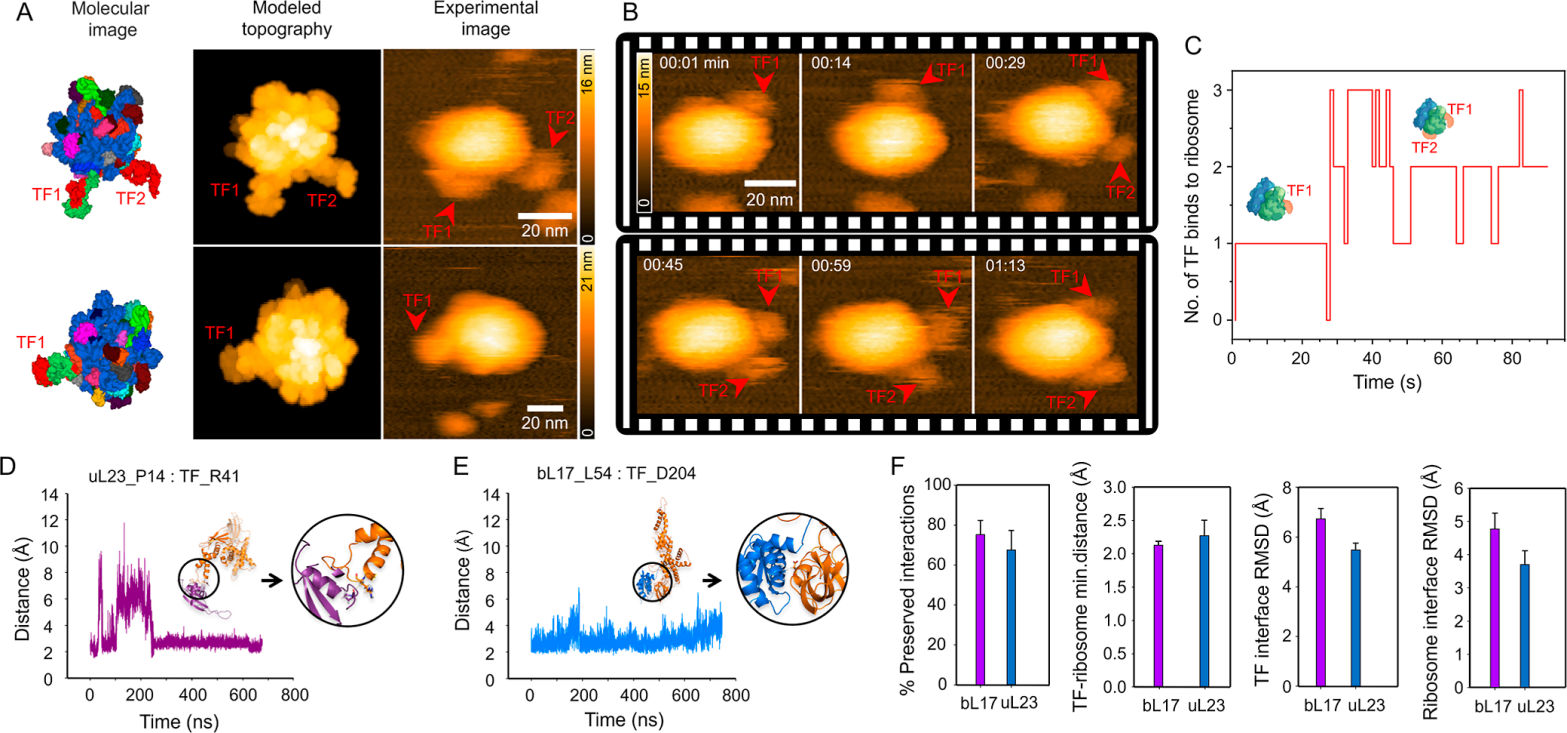
Multivalent binding of TF molecules to RNC. (A) HS-AFM images from two different instances are shown alongside molecular images (first panel) and AFM modeled topographies (second panel). In the experimental image, the TFs are marked with red arrowheads. Molecular images and modeled topographies are based on the 50S ribosomal subunit (PDB: 6PJ6), with the NC predicted by AlphaFold2 (green) and TFs (red). (B) Sequential frames showing two TF molecules (indicated with red) dynamically binding to an RNC. Time stamp is shown at the upper-left corner of each frame. (C) A plot showing the number of TF molecules bound over time in (B), capturing the frequency and duration of binding events. (D) Interaction of ribosomal protein uL23 with TF, analyzed by MD simulations. Inset: schematic representation of the interaction between uL23 and TF, highlighting uL23_P14 and TF_R41 residues. Plot showing the distance between these residues over ∼700 ns of simulation. (E) Interaction of ribosomal protein bL17 with TF as obtained from MD simulations. Inset: schematic representation of the interaction between bL17 and TF, highlighting bL17_D54 and TF_D204 residues. Plot showing the distance between these residues over ∼800 ns of simulation. (F) Plots summarizing the percentage of preserved bL17–TF and uL23–TF interactions, distances between these protein pairs, RMSD of the TF interface, and RMSD of the ribosomal protein interface, respectively.

Dwell time analysis showed that the exit-tunnel-bound TF exhibited a mean interaction time of 8.0 ± 1.1 s, with some events persisting for up to 80 s (Figure S3C). Since ribosomal protein uL23 is present near the nascent chain and known to provide the key docking site,^14,15^ the exit-site population likely comprises a mixture of TF bound to the NC alone, to uL23 alone, or concurrently to both NC and uL23. Looking at the survival curve, there is a possible deviation from a single-exponential fit in the ∼20–40 s range (Figure S3C), and the long tail of the distribution suggests the presence of a longer-lived subpopulation compatible with multivalent (NC + uL23) or sequential binding events of TF, rather than a single-step dissociation process. In contrast, peripheral TF interactions were shorter, averaging 3.9 ± 0.4 s, in some instances extending beyond 30 s (Figures S3C and S5, Videos S13, S14). These in vitro lifetimes are consistent with prior biochemical measurements of TF association with translating ribosomes, which report mean lifetimes of ∼10 s and occasional long-lived interactions up to ∼35–110 s depending on nascent chain length and hydrophobicity.^56,64^ Notably, in vivo single-molecule studies in *E. coli* have reported substantially shorter residence times (∼0.1–0.2 s),^75,76^ likely reflecting molecular crowding, transient sampling, and rapid dynamic exchange in the cellular environment. Therefore, the absolute dwell times observed by HS-AFM differ from cellular measurements but align with in vitro expectations and reveal site-specific functional asymmetry, where the exit-tunnel-bound TF serves as a stable anchor, while the peripheral TF transiently interacts with the neighboring ribosomal regions. In all observed events, the first TF binds near the exit-tunnel vestibule prior to the binding of a second TF at the peripheral ribosomal surface. This sequence may be obligatory or may simply reflect the higher affinity and stability of the vestibule interaction. This initial binding may involve direct interaction with the nascent chain, with uL23 or both, but the current data set does not allow these contributions to be distinguished unambiguously.

HS-AFM imaging revealed that TF engages near the ribosomal exit tunnel but lacks the spatial resolution to resolve individual receptor sites. To characterize these interactions at submolecular detail, we performed rigid-body docking of TF onto seven candidate ribosomal proteins (uL2, uL4, uL6, bL17, uL23, uL24, uL28), selected based on prior experimental evidence from proximity labeling, cross-linking, or cryo-EM mapping^14,61,64,77^ (Figure S6A). The highest-scoring docking models, selected by contact geometry and binding energy, served as starting points for 500 ns all-atom MD simulations to evaluate the interaction stability. Stability was assessed by (i) interfacial contact retention, (ii) minimum TF–protein distance, and (iii) RMSD of both TF and the ribosomal interface (Figure S6B). TF–bL17 and TF–uL23 exhibited the most stable interactions, with high contact retention (∼75.1% and ∼67.5%, respectively), close proximity (∼2.13 and ∼2.27 nm), and notable TF rearrangements (RMSD ∼6.7 and ∼3.7 Å). Both proteins are positioned at the tunnel vestibule and are implicated in cotranslational regulation; uL23 serves as a major docking site for TF, SRP, and SecYEG, while bL17 was identified as a TF partner via cross-linking.^61^ The TF–uL24 complex showed intermediate stability (70.7% contact retention, 3.02 nm distance, RMSDs of 2.95 Å and 3.14 Å), suggesting a more transient binding. In contrast, TF–uL2 and TF–uL28 displayed weak engagement, with a low contact retention (<45%) and an increased separation (>3 nm). TF interactions with uL4 and uL6, which lie farther from the vestibule, were negligible and thus excluded from further analysis.

Based on these findings, we conducted extended MD simulations (∼800 ns) on the TF–bL17 and TF–uL23 complexes to study their long-term stability and local interaction dynamics (Figure 4D,E; Videos S15 and S16). TF remained within sub-5 Å proximity of both ribosomal proteins. At the vestibule, the RBD helix of TF (residues R41 and D43) formed a persistent salt bridge with the uL23 backbone (residues P14 and V16), maintaining an interface RMSD of 3.7–4.0 Å. Concurrently, the PPIase loop (residues D204–G208) contacted the bL17 α-helix (residues L54–A61) via hydrophobic stacking and hydrogen bonding, albeit with greater flexibility (RMSD: 6.7–7.0 Å). Interaction persistence was ∼67% for uL23 and ∼75% for bL17 (Figure 4F), confirming stable and functionally relevant binding. TF binding induced only localized surface rearrangements of uL23 and bL17 (RMSD: 5.5–5.8 Å) without perturbing overall ribosome architecture, consistent with TF’s role as a peripheral, noninvasive chaperone. This structural plasticity may enable TF to support cotranslational folding while dynamically exchanging with other factors at the exit tunnel.^15,50,51,56,78^ Additionally, the observed PPIase–bL17 interaction is consistent with previous reports suggesting that the PPIase domain of TF can play a noncatalytic, auxiliary role in ribosome association and substrate handling.^20,79^

Consistent with MD results, structural comparison between the empty ribosome (PDB: 7K00 ^35^) and RNC + TF complex (PDB: 8ZFI^80^) indicates that TF binding largely preserves the global ribosomal architecture while introducing localized flexibility in bL17. bL17 maintains its overall fold yet shows decreases in model-to-density agreement (negative Δ*Q*) and increased *B*-factors in specific hydrophobic regions, including the central loop (Phe80, Leu83), a hydrophobic patch near residues 51–53 (Leu51, Ile52), and the β-sheet–adjacent region around Cys100 and Phe102 (Figure S7). These changes are more pronounced and spatially confined than in uL16, a more distal protein used here as an internal control, suggesting that TF engagement subtly destabilizes discrete hydrophobic patches. Such local rearrangements may facilitate transient TF interactions without compromising global ribosomal integrity (a detailed per-residue Δ*Q* and *B*-factor analyses in Supplementary Results and in Figure S7).

## Conclusions

In this study, we integrated HS-AFM imaging and all-atom MD simulations to unravel the real-time behavior and multivalent interactions of the bacterial chaperone TF on the 70S RNC. HS-AFM imaging under near-physiological conditions revealed that TF monomers dynamically interconvert between extended, semicompact, and compact conformations, with AR distributions closely mirrored by MD-derived conformations. Notably, we observed that two TF molecules can engage in a single RNC, forming both long-lived contacts at the exit-tunnel vestibule (near uL23) and more transient interactions on the ribosomal periphery (near bL17). These findings challenge the prevailing 1:1 TF–ribosome interaction model and support a cooperative, multivalent recruitment mechanism that is likely to be effective for substrate engagement during cotranslational folding.

Our MD simulations validated TF conformations and elucidated a dual-site binding mechanism. A persistent salt bridge between the RBD helix of TF (R41–D43) and the backbone of uL23 (P14–V16), along with hydrophobic and hydrogen bonding interactions between the PPIase loop (D204–G208) and bL17, collectively reveals a dual-site binding mechanism that enables both robust ribosomal association and conformational plasticity. Comparative analysis of high-resolution Cryo-EM structures revealed that binding of TF to uL23 allosterically increases flexibility in specific bL17 hydrophobic regions. This ″dynamic allostery″ creates transient docking sites that facilitate secondary interactions, structurally explaining the ∼ 4s binding events observed by HS-AFM. TF binding induced only local rearrangements without perturbing overall ribosome architecture, reinforcing its role as a peripheral, noninvasive chaperone capable of accommodating diverse nascent sequences without disrupting translational function.

We acknowledge that the use of stalled translation intermediates may stabilize interactions that are more transient during active elongation. In vivo, continuous nascent chain elongation and dynamic factor exchange likely modulate the TF binding kinetics and residence times. Therefore, real-time analyses under processive translation conditions will be critical to fully elucidate the kinetics and regulation of cotranslational folding. Future studies combining site-directed mutagenesis of the identified TF–bL17 interface with biochemical and in vivo functional assays will be essential to establishing the physiological role of this newly identified interaction.

Looking ahead, our work establishes a platform for real-time, in situ studies of cotranslational folding and chaperone dynamics using HS-AFM. This approach is readily extendable to other ribosome-associated factors such as the signal recognition particle (SRP) and elongation factors. Future studies performed under processive translation conditions and with site-specific functionalization strategies will be essential to capture short-lived, nascent chain-driven interactions and post-translational modifications with higher temporal precision. Ultimately, this integrative approach may open new avenues for directly visualizing ribosomes in action, mapping the kinetic and structural landscape of the translational chaperone network, and exploring targeted interventions in protein-folding diseases.

## Materials and Methods

### Ribosome 70S Purification

*E. coli* cells were grown in LB at 37 °C to OD_600_ ∼0.6, harvested (5000*g*, 10 min, 4 °C), washed in PBS, and lysed in 20 mM Tris-HCl (pH 7.5), 10 mM MgCl_2_, 100 mM NH_4_Cl, 0.5 mM EDTA, and 6 mM β-mercaptoethanol by high-pressure homogenization. Clarified lysate (30,000*g*, 30 min, 4 °C) was washed with 500 mM NH_4_Cl, layered onto 30% sucrose, and centrifuged at 100,000*g* for 16 h. The pellet was resuspended in low-salt buffer, applied to a 10–40% sucrose gradient, and centrifuged at 150,000*g* for 5 h. Fractions corresponding to 70S were pooled, analyzed by SDS-PAGE, aliquoted, and stored at −80 °C.

### Trigger Factor Purification

His-tagged TF was expressed in *E. coli* BL21­(DE3) with 0.5 mM IPTG induction at 25 °C for 4 h. Cells were sonicated in 50 mM Tris-HCl (pH 7.5), 300 mM NaCl, 10 mM imidazole, and 1 mM PMSF; lysate was clarified (30,000*g*, 30 min, 4 °C). Supernatant was applied to Ni-NTA resin, washed (20 mM imidazole), eluted (250 mM imidazole), then dialyzed into 20 mM Tris-HCl (pH 7.5), 150 mM NaCl, 1 mM DTT, concentrated, and polished by Superdex 200. Fractions of pure TF were confirmed by SDS-PAGE and stored at −80 °C.

### HS-AFM Imaging

Ribosomes (5 nM) prepared in a buffer consisting of 120 mM KAc, 25 mM Tris pH 7.5, 14 mM MgAc, and 1 mM β-Me were partially immobilized onto the mica surface, followed by rinsing and finally imaging at 1 frame/s in tapping mode using USC-F1.2-k0.15 cantilevers (resonance frequency: 600 kHz in liquid) at 22–25 °C on an SS-NEX HS-AFM setup. TF (1 μM) was introduced into the imaging solution before scanning. Images were postprocessed in ImageJ via custom macros that primarily included noise reduction and background correction^81^ (source code: https://github.com/centuri-engineering/ProtruDe/). AR were calculated in ImageJ (Feret Y/Feret X).

### Molecular Dynamics Simulations

The TF monomer (PDB: 1T11) was simulated with CHARMM36/TIP3P and Amberff19SB/OPC in GROMACS 2020 and Amber 22. After minimization and equilibration (NVT/NPT), production runs (2.5–3.3 μs per replica) were performed at 300 K with PME simulated electrostatics (1.2 Å grid, 9 Å cutoff). The RNC–TF complex from AlphaFold Multimer was simulated in NAMD 3.0 with CHARMM36/TIP3P for 500 ns × 3 replicas; interface distances were monitored. TF docking to individual ribosomal proteins (PDB: 7K00) was carried out in Piper, refined by 100 ns GROMACS simulations, and analyzed for contact stability (RMSD, center-of-mass distances, contact preservation).

### Modeled AFM Images

The modeled AFM images were generated by using BioAFMviewer software (version 4.1) to have an estimate of the molecular configurations observed in our experimental HS-AFM images. The following parameters were used for the simulated scanning: a probe tip radius of 1 nm and a cone angle of 5°.

Further details are provided in the Supporting Information.

## Supplementary Material



## Data Availability

The data sets analyzed and used in this study are available with the manuscript and in the Supporting Information.

## Notes

Extended materials and methods; table S1: details of the molecular dynamics simulations; supplementary results: conformational transitions of TF revealed by MD simulations, and detailed Δ*Q* and *B*-factor analysis of uL16 and bL17 in empty and TF/nascent chain-bound ribosomes; supplementary figures: population distribution and structural dynamics of 70S ribosome, TF monomer and dimer distribution and aspect ratio variation, dynamics of TF interactions with ribosomes, dynamics of the interaction between TF and the nascent chain based on all-atom MD simulations, multivalent binding of TFs to RNC, screening of TF interactions with vestibule-adjacent ribosomal proteins based on MD simulations, and comparative analysis of uL16 and bL17 between ribosome alone (PDB: 7K00) and TF/nascent chain-bound (PDB: 8ZFI) ribosome; supplemental video information; and description of datasets (PDF)

## ¶

Institute of Physics, University of Augsburg, Augsburg, Germany
