# Supplementary material for: Multivalent Interactions between Chaperone and Ribosome-Nascent Chain Complex Revealed by High-Speed AFM and MD Simulations
Source: ACS Nano. 2025 Dec 11;19(50):42275–87. doi: 10.1021/acsnano.5c13500 (PMC12752688; doi:10.1021/acsnano.5c13500)
Supplement: Supplementary file 1 [file nn5c13500_si_001.pdf]

Supporting Information for

Multivalent Interactions between Chaperone and Ribosome-Nascent Chain Complex Revealed by High-Speed AFM and MD Simulations

Eider Nuñez*,▽, Prithwidip Saha▽, Markel G. Ibarluzea, Arantza Muguruza-Montero, Sara M-Alicante, Rafael Ramis, Aritz Leonardo, Aitor Bergara, Alvaro Villarroel, and Felix Rico*.

▽ Equal contributions

*Email: eider.nunez@ehu.eus and felix.rico@inserm.fr

This PDF file includes:

Extended Materials and Methods

Table S1

Supplementary Results

Supplementary Figures 1 to 7

Supplemental Video Information 1 to 16

Description of Datasets

References for SI

Other supporting materials for this manuscript include:

Videos S1 to S16

Datasets S1 to S5

EXTENDED MATERIALS AND METHODS

1. Sample expression and purification

a. 70S ribosome purification from *E. coli*

To purify 70S ribosomes from *Escherichia coli*, cells were cultured in LB medium (Thermo Fisher Scientific, #12780052) at 37 °C until reaching an OD_{600} of 0.5–0.7, corresponding to mid-logarithmic growth. Cells were harvested by centrifugation at 5,000 × g for 10 minutes at 4 °C to maintain ribosome integrity. The resulting pellets were washed with ice-cold PBS (Gibco, #70011036) and stored on ice until lysis.

Bacterial pellets were resuspended in lysis buffer (20 mM Tris-HCl, pH 7.5, 10 mM $MgCl_2$, 100 mM NH_4Cl, 0.5 mM EDTA, and 6 mM β-mercaptoethanol; Sigma-Aldrich, #M6250) and lysed using a high-pressure homogenizer (Avestin, EmulsiFlex-C5). The lysate was clarified by ultracentrifugation at 30,000 × g for 30 minutes at 4 °C to remove unlysed cells and large debris.

The resulting supernatant was subjected to a high-salt wash with buffer containing 20 mM Tris-HCl (pH 7.5), 500 mM NH_4Cl, 10 mM $MgCl_2$, and 6 mM β-mercaptoethanol to eliminate loosely associated proteins. The washed supernatant was layered onto a 30 % sucrose cushion prepared in ribosome stabilization buffer (20 mM Tris-HCl, pH 7.5, 10 mM $MgCl_2$, 100 mM NH_4Cl, and 6 mM β-mercaptoethanol) and centrifuged at 100,000 × g for 16–18 hours at 4 °C using a Beckman Coulter ultracentrifuge with a SW32 Ti rotor.

The ribosome pellet was carefully resuspended in low-salt buffer (20 mM Tris-HCl, pH 7.5, 10 mM $MgCl_2$, 50 mM NH_4Cl, and 6 mM β-mercaptoethanol) to avoid aggregation. For further purification, the resuspended pellet was layered onto a 10–40 % sucrose gradient in the ribosome stabilization buffer and ultracentrifuged at 150,000 × g for 5 hours at 4 °C.

Ribosomal fractions were collected by monitoring absorbance at 260 nm to identify the 70S ribosome peak. Collected fractions were analyzed by SDS-PAGE (NuPAGE™ 4–12 % Bis-Tris Protein Gels, Invitrogen, #NP0321BOX) to confirm purity. Purified ribosomes were aliquoted and stored at –80 °C.

b. Ribosome-nascent chain (RNC) purification

E. coli BL21(DE3) cells were transformed with the plasmid pProEx-htC encoding the nascent chain construct mtfp–hAB–SecM–mcpVenus. Transformants were plated on selective LB agar, and colonies exhibiting fluorescence were selected for further culture.

Selected colonies were grown in LB medium (Thermo Fisher Scientific, #12780052) at 37 °C until reaching an OD600 of 0.6, at which point expression was induced with 0.3 mM IPTG. Cultures were incubated for 1 h at 37 °C and harvested by centrifugation at 5,000 × g for 10 min at 4 °C. Pellets were washed with ice-cold PBS (Gibco, #70011036) and stored on ice until lysis.

Cells were resuspended in lysis buffer (20 mM Tris-HCl, pH 7.5; 10 mM $MgCl_2$; 100 mM NH_4Cl; 0.5 mM EDTA; 6 mM β-mercaptoethanol; Sigma-Aldrich, #M6250) and lysed using a high-pressure homogenizer (Avestin, EmulsiFlex-C5). The lysate was clarified by ultracentrifugation at 30,000 × g for 30 min at 4 °C.

The clarified supernatant was layered onto a 30 % sucrose cushion in ribosome stabilization buffer (20 mM Tris-HCl, pH 7.5; 10 mM $MgCl_2$; 100 mM NH_4Cl; 6 mM β-mercaptoethanol) and centrifuged at 100,000 × g for 16–18 h at 4 °C. The resulting pellet was resuspended and further purified through a 10–40 % sucrose gradient at 150,000 × g for 5 h at 4 °C to isolate intact ribosomal particles.

To specifically isolate RNCs from free 70S ribosomes, gradient fractions were subjected to nickel-affinity chromatography, exploiting the N-terminal His-tag on the nascent chain. Bound RNCs were washed and eluted under native conditions, yielding highly pure complexes suitable for subsequent HS-AFM imaging.

Arrested sequence: RNC (SecM) (Protein)

MSYYHHHHHHDYDIPTTENLYFQGAMASVSKGEETTMGVIKPDMKIKLKMEGNVNGHAFVIEGE
GEGKPYDGTNTINLEVKEGAPLPFSYDILTTAFAYGNRAFTKYPDDIPNYFKQSFPEGYSWERTMT
FEDKGIVKVKSDISMEEDSFIYEIHLKGENFPPNGPVMQKKTTGWDASTERMYVRDGVLKGDVKH
KLLLEGGGHHRVDFKTIYRAKKAVKLPDYHFVDHRIEILNHDKDYNKVTVYESAVARNSTDGMD
ELYKILGSGFALKVQEQHRQKHFEKRRNPAAGLIQSAWRFYATNLSRTDLHSTWQYYEFTVTVPM
YREDLTPGLKVSIRAVCVMRFLVSKRKFKESLREFYVGYVPGGSPGRPGGSRPHVGSGGQQGSHV

FSTPVWISQHAPIRGSPGSSVDMYSDGGVQLADHYQQNTPIGDGPVLLPDNHYLSYQSKLSKDPNE
KRDHMVLLEFVTAAGITLGMDELYKGGSGGMVSKGEELFTGVVPILVELDGDVNGHKFSVSGEG
EGDATYGKLTLKLICTTGKLPVPWPTLVTTLGYGLQCFARYPDHMKQHDFFKSAMPEGYVQERTI
FFKDDGNYKTRAEVKFEGDTLVNRIELKGIDFKEDGNILGHKLEYNYNSHNVYITADKQKNGIKA
NFKIRHNIEPSMLEACGTKLGCFGG

mTFP1

α-helix_1

α-helix_2

α-helix_3

SecM

mcpVenus

c. Trigger factor purification

Trigger Factor (TF) protein was purified from *E. coli* strain BL21 (DE3) cells (Novagen, #69450) transformed with a plasmid encoding His-tagged TF at the N-terminal. Cultures were grown in LB medium (Thermo Fisher Scientific, #12780052) supplemented with 50 μg/mL kanamycin (Sigma-Aldrich, #K0254) at 37 °C to an OD_{600} of 0.6–0.8. Protein expression was induced by adding 0.5 mM IPTG (Thermo Fisher Scientific, #15529019), and cultures were incubated for an additional 4 hours at 25 °C to optimize protein folding.

Cells were harvested by centrifugation at 5,000 × g for 15 minutes at 4 °C, and the pellet was washed with ice-cold PBS (Gibco, #70011036). The bacterial pellet was resuspended in lysis buffer (50 mM Tris-HCl, pH 7.5, 300 mM NaCl, 10 mM imidazole, and 1 mM PMSF) and lysed by sonication on ice with a Qsonica sonicator (model Q500) using 10 cycles of 10 seconds on, 20 seconds off. The lysate was clarified by centrifugation at 30,000 × g for 30 minutes at 4 °C.

The supernatant was loaded onto a Ni^{2+}-NTA affinity column (HisTrap HP, GE Healthcare, #17-5248-01) pre-equilibrated with a lysis buffer. Unbound proteins were removed by washing the column with a wash buffer (50 mM Tris-HCl, pH 7.5, 300 mM NaCl, 20 mM imidazole) until baseline absorbance returned to zero. TF was then eluted with an elution buffer containing 50 mM Tris-HCl, pH 7.5, 300 mM NaCl, and 250 mM imidazole (Sigma-Aldrich, #I5513).

Eluted fractions were dialyzed overnight at 4 °C against buffer (20 mM Tris-HCl, pH 7.5, 150 mM NaCl, 1 mM DTT) using a dialysis membrane with a 10 kDa cutoff (Thermo Fisher Scientific, #68100). Following dialysis, the sample was concentrated with Amicon Ultra-15 Centrifugal Filters (Millipore, #UFC901008) and further purified by size-exclusion chromatography on a Superdex 200 column (GE Healthcare, #28990944) equilibrated with a buffer (20 mM Tris-HCl, pH 7.5, 150 mM NaCl, 1 mM DTT).

Eluted fractions containing pure TF were confirmed by SDS-PAGE using NuPAGE™ 4–12 % Bis-Tris gels (Invitrogen, #NP0321BOX) and stained with Coomassie Brilliant Blue (Bio-Rad, #1610436). The purified TF fractions were pooled, concentrated, and stored at –80 °C in aliquots for subsequent analysis.

2. HS-AFM imaging

a. Sample preparation

A freshly cleaved muscovite mica disc (diameter: 1.5 mm, thickness: 0.1 mm, JBG-Metafix, Montdidier, France) was used as a surface without any further modification. The mica was then glued to a glass stage for imaging purposes. Ribosomes were prepared in an imaging buffer consisting of 120 mM potassium acetate (KAc), 25 mM Tris (pH 7.5), and 1 mM β-mercaptoethanol (β-Me), supplemented with 14 mM magnesium acetate (MgAc). A 2 μL aliquot of the resulting solution, at a concentration of 5 nM, was deposited onto the mica surface and incubated for 2–3 minutes to allow for partial immobilization of the ribosomes under native-like buffer conditions. Magnesium ions (Mg^{2+}) stabilize the ribosomal structure by shielding negative charges on rRNA and maintaining subunit integrity, while also mediating electrostatic interactions with the negatively charged mica surface. Given the relatively uniform net surface charge of the ribosome, adsorption in multiple

orientations is equally likely, leading to the variety of views observed in the HS-AFM data. Following incubation, the surface was washed several times with the imaging buffer to remove any unbound ribosomes. HS-AFM imaging was conducted in the same buffer environment, with ~100 µL of buffer in the imaging chamber. For experiments involving TFs, it was introduced directly to the imaging chamber at a final concentration of 5 nM, followed by an incubation period of 10 minutes before imaging.

b. Data acquisition

Imaging was conducted in tapping mode on an SS-NEX HS-AFM equipped with a standard scanner and ultrashort cantilevers having a resonance frequency of 600 kHz in liquid and a nominal spring constant of 0.15 N/m (USC-F1.2-k0.15, NanoWorld, Neuchâtel, Switzerland). During scanning, the free oscillation amplitude of the cantilever was set to 3–4 nm, and the set point for feedback control was kept 20 % lower than this value. Images were acquired at a scan rate of 1 frame per second. Typically, the pixel size was either 0.33 or 0.5 nm per pixel, while the typical size of a scan area was 300 × 300 nm^2. At least 3 independent experiments were performed both for the ribosome alone and the ribosome in the presence of TF. All measurements were performed at room temperature (22–25 °C) in the imaging buffer.

c. Image processing and analysis

All HS-AFM videos were processed using in-house developed macros implemented in Fiji (ImageJ) image processing software to semi-automate the image processing. The processing steps mainly involved noise reduction, background correction (tilt correction), and the removal of unwanted background particles. To detect the binding of TF to the ribosome, we employed another macro to identify the bound TF as a protrusion on the ribosomal surface across hundreds of images. The code used for image processing is available on the GitHub repository.[1] The LAFM map was generated upon applying a localization algorithm on HS-AFM raw data, available as an ImageJ plugin.[2–4]

The length-to-width aspect ratio was calculated using ImageJ by dividing the FeretY (the maximum caliper length) by the FeretX (the maximum caliper width).

3. P-stalk angle measurement

To determine P-stalk angle, we extracted three cross-sectional profiles from each AFM frame and identified local maxima corresponding to the ribosome center (R_{center}), stalk center (S_{center}) and stalk tip (S_{tip}). The coordinates of the local maxima correspond to the pixel positions with maximum height. The angle θ at S_{center} was determined using the positions of the three local maxima: R_{center}, S_{center}, and S_{tip}. To estimate uncertainty, we bootstrapped the positions by sampling neighboring pixels (±1 pixel) 1000 times and reported the mean ± SD for θ (Figure S1C).

4. MD simulations

a. TF monomer simulations

MD simulations of the TF monomer (PDB: 1T11) were performed using two force fields: CHARMM36[5] with TIP3P water[6] and Amber ff19SB[7] with OPC water.[8] The CHARMM36 simulations were conducted using GROMACS 2020,[9] while Amber simulations were carried out using the pmemd program in Amber2270.[10]

The TF monomer was placed in a cubic simulation box with periodic boundary conditions and solvated in a cubic box with an ionic concentration of 0.15 M NaCl. For the CHARMM36 systems, the preparation included energy minimization, followed by 500 ps each of NVT and NPT equilibration. For the Amber systems, two NPT equilibration steps followed the initial NVT equilibration: a 100 ps equilibration with a 1 fs timestep and a subsequent 1 ns equilibration with a 2 fs timestep. These simulations were performed at 300 K using Langevin dynamics. Long-range electrostatics were computed using the particle-mesh Ewald (PME) method, with a grid spacing of 1.2 Å,[11] and a nonbonded cutoff of 9 Å was applied.

Production simulations were conducted for both force fields with two replicas per system. The total simulated times were 2.67 µs and 2.56 µs for CHARMM36 systems, and 3.25 µs and 3.06 µs for Amber systems (Table 1). The solvent-accessible surface area (SASA) of the TF protein was computed for all simulations using the gmx sasa program.

For all the simulations, VMD software[12] was used for analysis of the trajectories and generation of certain figures shown in this study. PyMol software (Pymol) was used to render figures 3D, 4D, S6A and S7A.

b. TF binding to RNC complex

The interaction between the RNC and TF was predicted using AlphaFold Multimer[13] and the model with the best ipTM + pTM score was prepared for simulation. Briefly, hydrogen atoms were added to the complex using the VMD software.[12] The system was then solvated resulting in a cubic box of dimensions described in Table S1. K^+ and Cl^- ions were added using the system resulting in an ionic concentration of 0.15 M KCl. The preparation included energy minimization, followed by NVT and NPT equilibration, first at 1 fs timestep for 250 ps each, and another 250 ps for the NPT simulation at 2 fs. Three individual replicas were run for 500 ns.

These simulations were performed with NAMD3.0.1.[14] The CHARMM36 force field[5] was used to model the protein and ions, and the TIP3P model[6] was chosen for the water. The PME method[11] was used for the treatment of electrostatic interactions. Electrostatic and van der Waals forces were calculated in every time step with a 12 Å cutoff distance. A switching distance of 10 Å was chosen to smoothly truncate the non-bonded interactions. The Nose-Hoover-Langevin piston method was employed to control the pressure with a 50 fs period, 25 fs damping constant, and a desired value of 1 atmosphere. These simulations were performed at 298 K using Langevin dynamics.

Distances between the RNC and the C-terminal of TF were measured by computing the minimum distance between pairs of heavy atoms in the interaction interface throughout the trajectories of the three replicas. This interaction interface was defined as residues 286 to 355 for the RNC and residues 300 to 392 for the TF. The same approach was used to compute minimum distances between residues 304–305 from RNC and residues 320 and 377 of TF.

c. Ribosome–TF docking and MD simulations

Rigid body docking between ribosomal proteins uL2, uL4, uL6, bL17, uL23, uL24, and uL28 and the TF monomer was performed using Piper.[15] The ribosomal proteins were extracted from the entire ribosome structure (PDB: 7K00) and docked in isolation with the TF monomer (PDB: 1T11). Some poses generated through docking were deemed unrealistic, as they positioned TF in regions inaccessible within the entire ribosome structure. Therefore, for each ribosomal protein, physically implausible poses were filtered from the top 15 poses generated.

MD simulations of the remaining poses were carried out using GROMACS 2020[9] with the CHARMM36 force field[5] (Table 1). The systems were solvated with TIP3P water molecules,[6] and neutralized to an ionic concentration of 0.15 M. The MD protocol consisted of four stages: energy minimization, NVT equilibration, NPT equilibration, and production.

During equilibration, a 500 ps NVT simulation at 300 K was performed using Langevin dynamics with a 2 fs timestep. Long-range electrostatics were treated using the PME method,[11] with a grid spacing of 1.2 Å, and a nonbonded cutoff of 12 Å. Positional restraints were applied to the protein alpha-carbons during this step. Following NVT equilibration, a 500 ps NPT equilibration was conducted at 1 bar using the Parrinello-Rahman barostat.[16] Production runs of 100 ns were performed for each system starting from the equilibrated structures.

The simulations were analyzed to characterize the binding interfaces, defined as residues within 6 Å of the opposing protein. Metrics included the percentage of preserved contacts relative to the final frame, the distance between the centers of mass of both interfaces, and the RMSD of the ribosomal and TF interfaces. These metrics were used to assess stability and identify unbinding events or instability in docking-predicted poses. Specifically, a significant increase in the distance between the centers of mass indicated unbinding, while slight decreases reflected interface rearrangements resulting in more stable conformations not captured during docking. Poses with stable center-of-mass distances but low contact preservation or high RMSDs were also considered unstable.

The results of these initial simulations served as an additional filter to select the most stable docking poses. Stable poses were subjected to extended simulations of ~800 ns each to validate their stability further. The metrics from these extended simulations were used to rank the remaining poses, and the most stable one for each ribosomal protein was used as the final model for analysis.

5. Alignment and comparison between PDB structures 7K00 and 8ZFI

Two high-resolution ribosome structures (PDB IDs: 7K00 and 8ZFI) were aligned using the MatchMaker tool in ChimeraX,[17] with alignment performed based on the uL16 and bL17 ribosomal proteins. Following alignment, per-residue root-mean-square deviations (RMSDs) and secondary structure assignments based on the DSSP algorithm were computed using the mdtraj package.[18] Per-residue Q-scores[19] quantifying the agreement between atomic coordinates and electron density were calculated using the QScore tool in ChimeraX.

TABLE S1

Details of MD simulations

System (Forcefield)	Water model	# water molecules	Software	# atoms	Replicas	Simulation time (per replica)
TF monomer (CHARMM)	TIP3P	33737	Gromacs	108229	2	2680 ns
TF monomer (AMBER)	OPC	34373	Amber22	110205	2	3250 ns
TF + RNC (CHARMM)	TIP3P	108382	NAMD	337347	3	1000 ns
TF + bL17 (CHARMM)	TIP3P	26015	Gromacs	86167	1	800 ns
TF + uL23 (CHARMM)	TIP3P	36111	Gromacs	116098	1	800 ns

SUPPLEMENTARY RESULTS

1. Conformational transitions of TF revealed by MD simulations

MD simulations revealed three predominant conformational states of TF: an extended (E) conformation, a semi-compacted (SC) intermediate, and a compacted (C) form. The conformational transition from the E to the C state occurred through two largely independent interaction events that together drove the global collapse of TF.

The first event involved transient contacts between the PPIase domain and arm 1 of the C-terminal domain. These interactions were dynamic and reversible throughout the simulation. The second, more structurally significant event entailed the formation of a stable interface between the N-terminal domain and both arms 1 and 2 of the C-terminal domain. This interface represented the primary driver of TF's hydrophobic collapse.

Interestingly, this N–C-terminal interface could form with either C-terminal arm positioned between the N-terminal domain and the remaining arm, resulting in a layered arrangement. In such a configuration, one C-terminal arm simultaneously interacted with both the N-terminal helices and the second C-terminal arm. Once established, this interface remained stable throughout the remainder of the simulation.

By contrast, the PPIase domain continued to associate and dissociate from arm 1 dynamically, although it could occasionally form more persistent contacts once the N–C-terminal interface was already in place. These interactions contributed to the formation of fully collapsed conformations of TF.

2. Detailed ΔQ and B-factor analysis of uL16 and bL17 in empty and TF/nascent chain-bound ribosomes

Consistent with the MD results, comparative analysis of high-resolution structures (ribosome alone, PDB: 7K00; TF/NC-bound ribosome, PDB: 8ZFI) indicates that TF binding largely preserved the global ribosomal architecture. Structural alignment confirmed that bL17 maintained its overall fold with a global RMSD of ≈0.93 Å (Figure S7A).

To probe local flexibility, we performed per-residue RMSD analysis, which revealed increased deviations in surface-accessible loops of both bL17 and uL16, with particularly pronounced effects in bL17 (Figure S7B). This finding was corroborated by increases in normalized B-factors in the same regions, confirming enhanced thermal motion particularly in the central loop and C-terminal tail of bL17, while the control protein uL16 showed minimal changes (Figure S7C).

Further analysis revealed local decreases in model-to-density agreement (negative ΔQ) concentrated in specific hydrophobic regions of bL17: the central loop (residues ~79–83, including Phe80 and Leu83), a hydrophobic patch near residues ~51–53 (Leu51, Ile52), and the β-sheet–adjacent region around residue ~100 (Cys100, Phe102) (Figure S7D). When compared with uL16, used as an internal control, the effects in bL17 were stronger and more spatially confined. bL17 contains multiple hydrophobic residues exhibiting pronounced negative ΔQ values ($\Delta Q \leq -0.25$), including Val29, Ile33, Ile52, Ile75, Val76, Leu79, and Cys100, whereas uL16 showed fewer comparable perturbations (notably Val93, $\Delta Q \approx -0.268$) (Figure S7D).

Secondary structures were largely conserved in both states (Figure S7E), indicating that the observed changes represent local flexibility rather than global structural rearrangements. Mean ΔQ values were −0.1565 (SD 0.082; N =136) for uL16 and −0.1999 (SD 0.104; N =120) for bL17. The weak correlation between ΔQ and intrinsic hydrophobicity (Pearson $r \approx -0.19$ for uL16; $r \approx -0.25$ for bL17) indicates that hydrophobicity alone cannot explain the observed ΔQ distribution (Figure S7F).

Overall, these observations support a dynamic allostery model, in which initial TF engagement and/or nascent chain presence subtly destabilize discrete hydrophobic surfaces in bL17, transiently forming docking sites for secondary TF contacts. Such transient and weak hydrophobic interactions may underlie the short-lived (~4 s) TF binding events observed experimentally, allowing rapid association–dissociation cycles without compromising global ribosomal integrity. Alternative interpretations, including differences in local resolution or model refinement, cannot be excluded and warrant further experimental validation.

SUPPLEMENTARY FIGURES

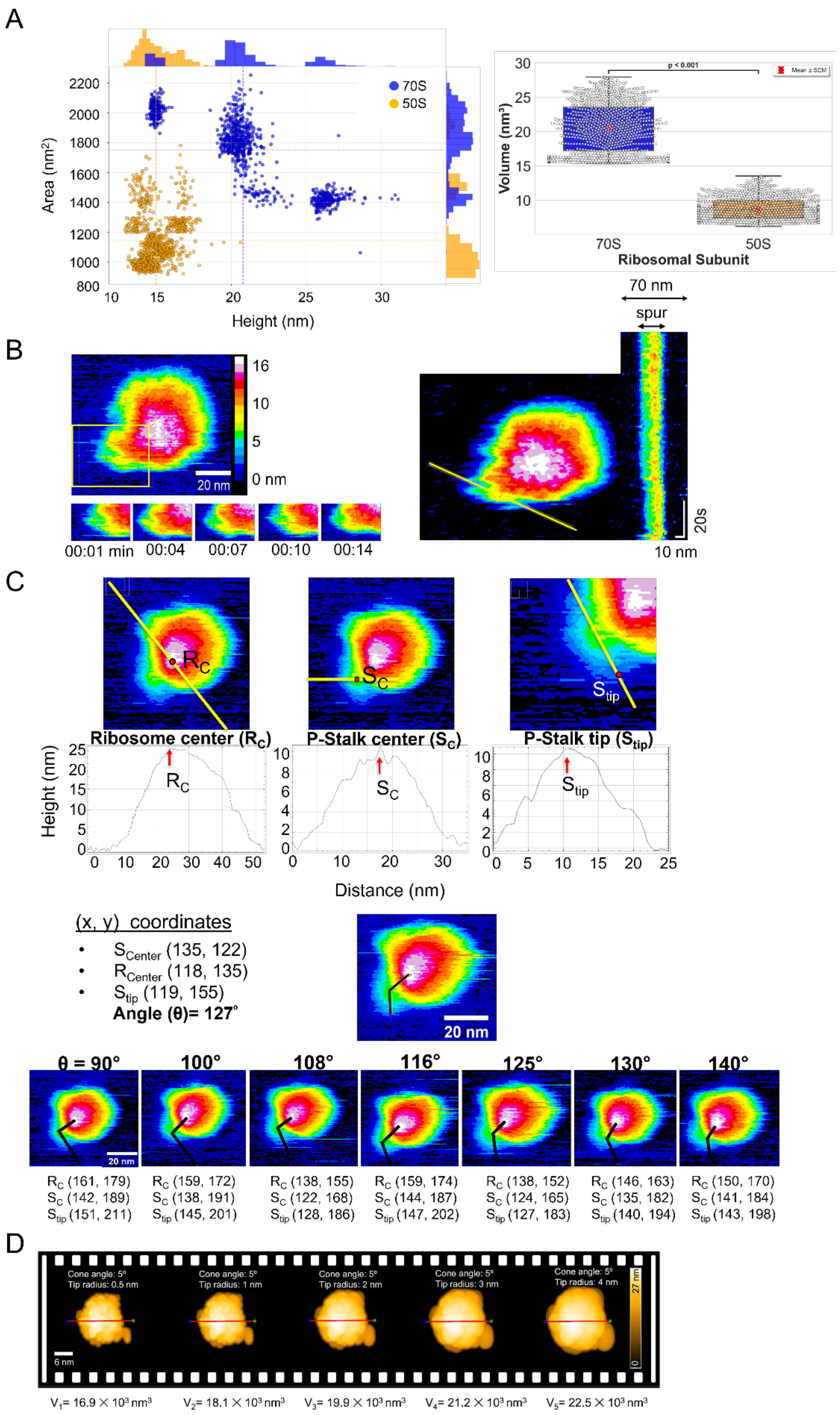

Figure S1. Population distribution and structural dynamics of 70S ribosome. (A) Left: Plot showing the relationship between ribosome height (x-axis) and area (y-axis) as measured by HS-AFM. The histograms above and to the right of the plot display the height and area distributions, respectively. Blue corresponds to the 70S ribosome (n =935), and orange represents the 50S subunit (n =935 frames). Right: Boxplots display volume distributions for 70S (blue) and 50S (orange) subunits (n =935 each). Boxes show interquartile ranges, central lines represent medians, whiskers indicate data range, and individual points show particle measurements. Red markers denote means ± SEM. ***$p < 0.001$ by t-test. **(B)** Left: Structural dynamics of the spur on the 30S subunit, with sequential frames highlighting the movement of the spur. These frames are a zoomed-in view of the region marked by a yellow rectangle in the extreme-left image. Right: A kymograph is shown, generated along the yellow line (spur) drawn across multiple HS-AFM frames. **(C)** Top panel: structural dynamics of the ribosomal P-stalk base of the 50S subunit in different AFM frames, with cross-sectional profiles shown with yellow lines. The angle (θ) was calculated using three reference points: the ribosome center, the P-stalk center, and the P-stalk tip. Local maxima for each reference point are highlighted with a red circle. Middle Panel: a reference frame showing the x, y coordinates used to compute the three points (image pixels) and the angle. Bottom panel: the angle (θ) between the proximal and distal regions of the P-stalk is indicated by a black line in the 7 frames from the same HS-AFM video. **(D)** Analysis of tip convolution effects. Modeled AFM topographs generated with BioAFMviewer using tip radii of 0.5, 1, 2, 3, and 4 nm (left to right) reproduce the lateral broadening in experimental images, caused by the finite tip size and the resulting increase in apparent volume.

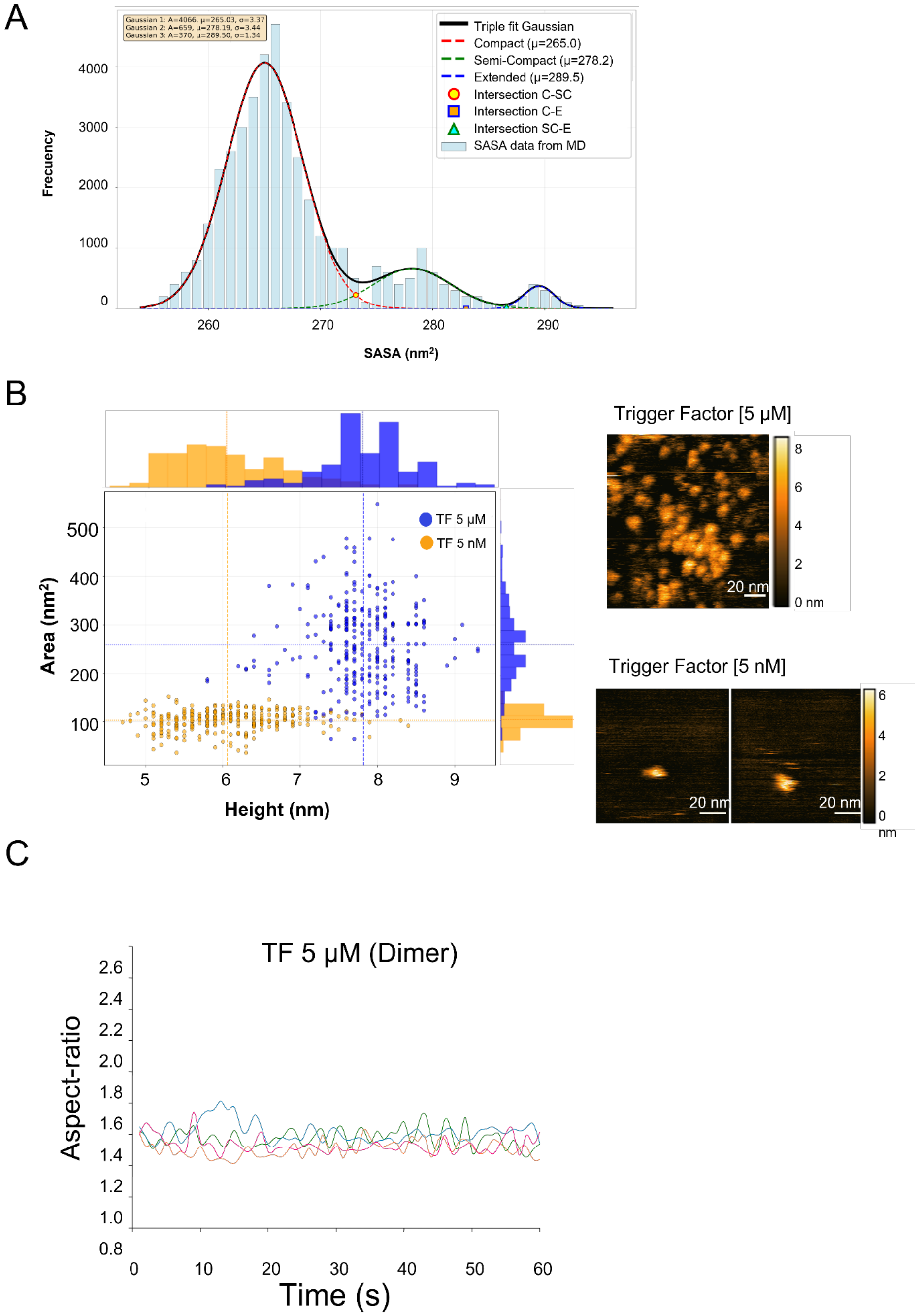

Figure S2. TF monomer and dimer distribution and aspect ratio variation. **(A)** Histogram of TF conformational states obtained from MD simulations based on their SASA values. The distribution was fitted using a Gaussian mixture model (R^2 =0.82), revealing three distinct populations: compact (SASA <273 nm^2; ~83 %), semi-compact (273≤ SASA ≤286; ~14 %), and extended (SASA > 286 nm^2; ~3.0 %). **(B)** Left: plot showing the relationship between the height and area of TF as measured by HS-AFM. The histograms above and to the right of the plot display the height and area distributions, respectively. Orange represents data from TF at 5 nM, and blue from TF at 5 µM. Top right: an AFM frame showing TF distribution on a mica surface at 5 µM concentration. Bottom right: two frames of TF at 5 nM concentration in different conformational states. **(C)** Time-dependent variation of AR of TF dimers during HS-AFM scanning. Data from different sets

of experiments are shown in distinct colors. Measurements were performed at a 5 μM concentration of TF in physiological buffer (25 mM Tris, pH 7.5, 120 mM KCl, 5 mM NaCl, 14 mM MgAc).

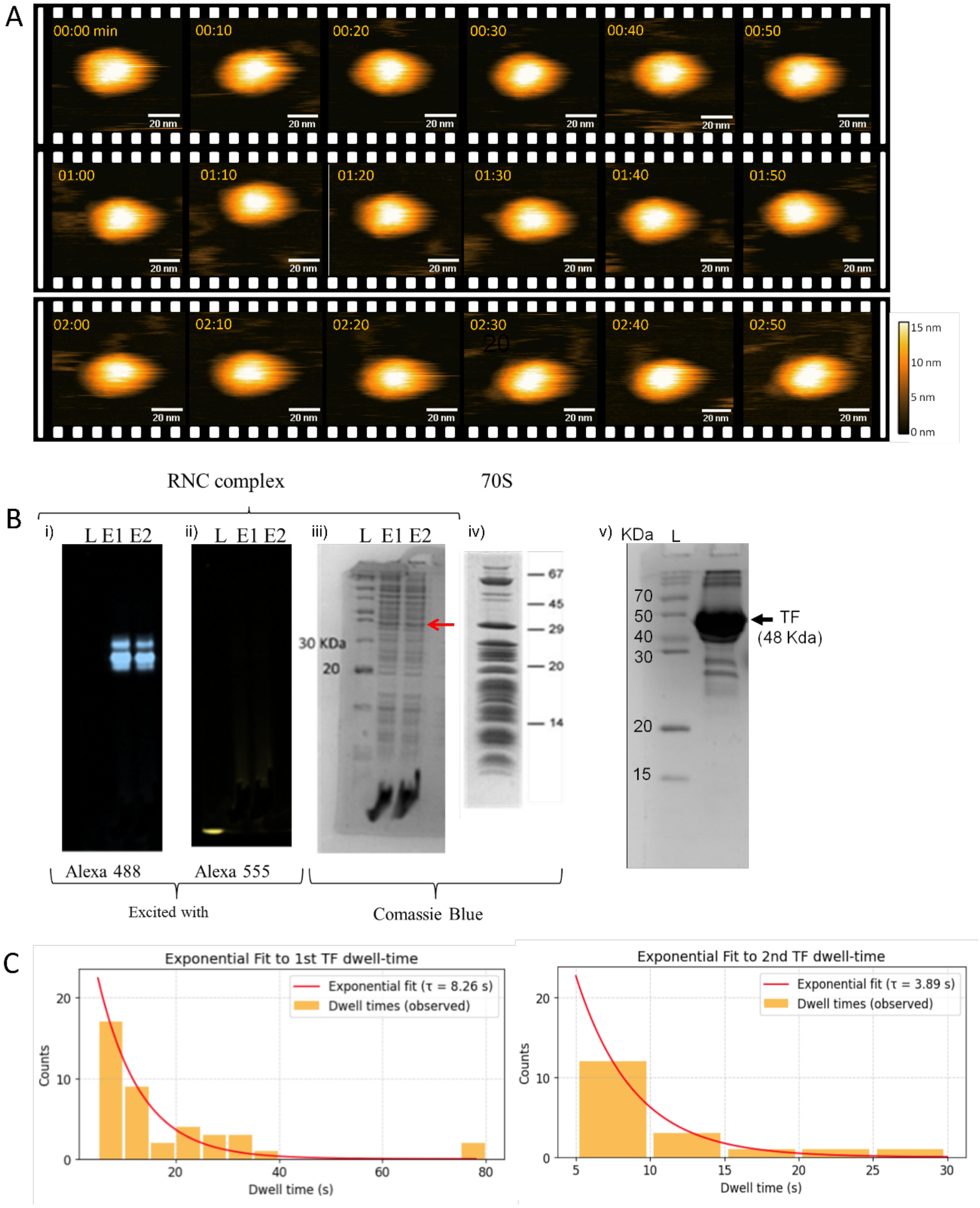

Figure S3. Dynamics of TF interactions with ribosomes. (A) Sequential frames from HS-AFM showing the 70S ribosome in the absence of a nascent chain and in the presence of 1 µM TF. There was no binding of TF to the ribosome detected. **(B)** Biochemical validation of nascent-chain arrest and complex integrity. **(i)** SDS–PAGE analysis (15% acrylamide) of the purified ribosome-nascent chain (RNC) complex. The designed construct features an N-terminal mTFP1 tag (cyan) and a C-terminal mcpVenus tag (yellow) separated by a SecM stalling motif. **(ii)** Fluorescence scan of the gel (488 nm excitation) showing a prominent band corresponding to the arrested full-length nascent chain. **(iii)** Fluorescence scan (555 nm excitation) of the same gel reveals no signal at the corresponding molecular weight, confirming the C-terminal mcpVenus remains untranslated due to efficient SecM-mediated stalling. **(iv)** Coomassie Brilliant Blue-stained gel with

the arrested nascent chain band indicated (red arrow). Lanes: L, protein ladder; E1 and E2, nickel-affinity elution fractions. **(v)** Coomassie-stained gel (15% acrylamide) of purified 70S ribosomes. **(f)** Coomassie-stained gel of purified Trigger Factor (TF), with the 48 kDa band indicated (black arrow). These data confirm efficient translational arrest and the integrity of the RNC complex, ensuring only the N-terminal domain is exposed for subsequent TF binding assays. **(C)** Top histogram shows the dwell time distribution of the first TF molecule interacting with RNC, with the red curve showing the exponential fit (mean interaction time τ $=8.3 \pm 1.1$ seconds). The bottom histogram corresponds to the dwell time distribution for a second TF molecule interacting simultaneously with the ribosome, for which the exponential fit yields a shorter time constant of $\tau = 3.9 \pm 0.4$ seconds.

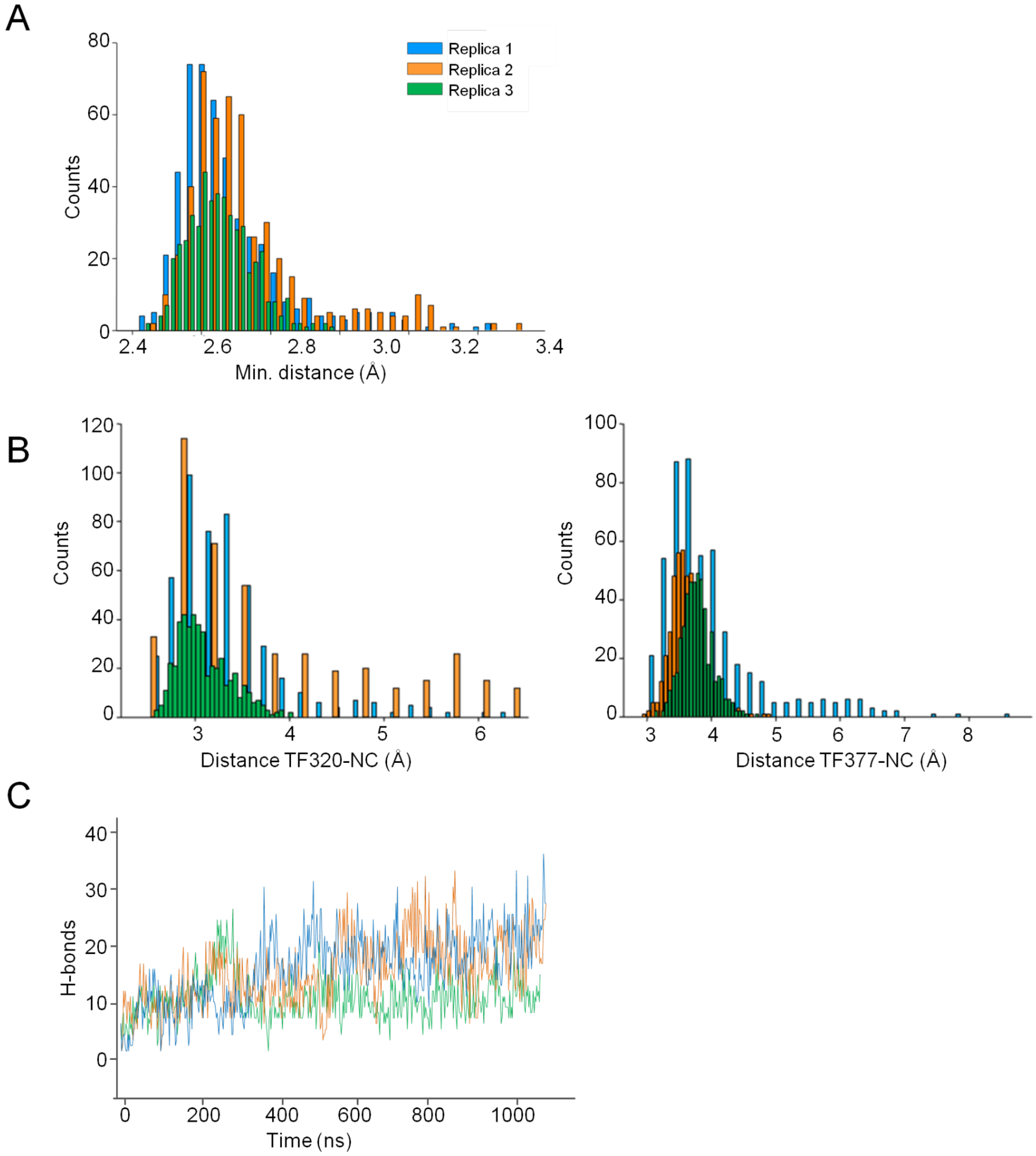

Figure S4. Dynamics of the interaction between TF and nascent chain, based on all-atom MD simulations. (A) Histogram showing the minimum distance between the C-terminal of TF (residues 300–392) and the hA-TW-B region of the nascent chain. Data from different replicates are shown in distinct colors. **(B)** Histogram showing the minimum distance between the TF320 (left) and TF377 (right) and the hA-TW-B region of the nascent chain. Data from different replicates are shown in distinct colors. **(C)** The number of hydrogen bonds between the two chains is shown across three replicates, with each replicate represented by a different color. The curves represent a moving average of the raw data.

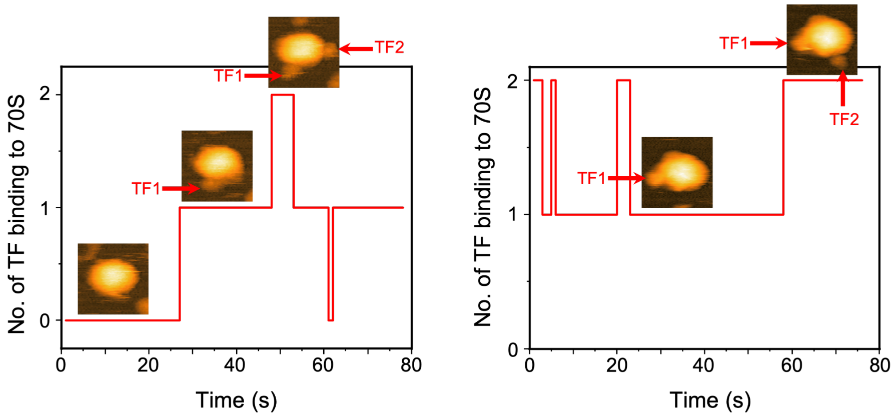

Figure S5. Multivalent binding of TFs to RNC. Plots show the binding trajectories of TF molecules upon interaction with the RNC as observed by HS-AFM, representing the frequency and duration of individual binding events. Data are presented from two independent HS-AFM videos, each capturing the RNC in a different orientation. Bound TF molecules are indicated with red arrows.

A

Ribosomal protein	Distance from exit tunnel (nm)
**L23** (Red)	1 - 1.5
**L24** (Orange)	1.5 - 2
**L4** (Pink)	4 - 5
**L17** (Cyan)	2.5 - 3.5
**L2** (Blue)	3 - 4
**L28** (Green)	4 - 5
**L6** (Purple)	10 - 12

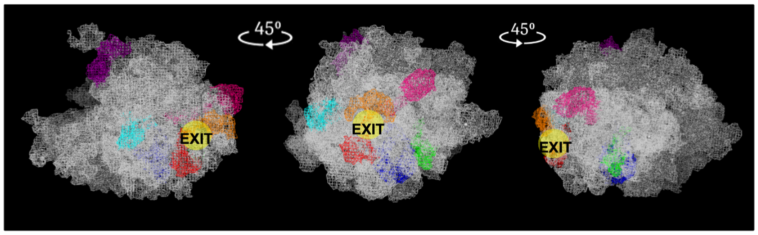

B

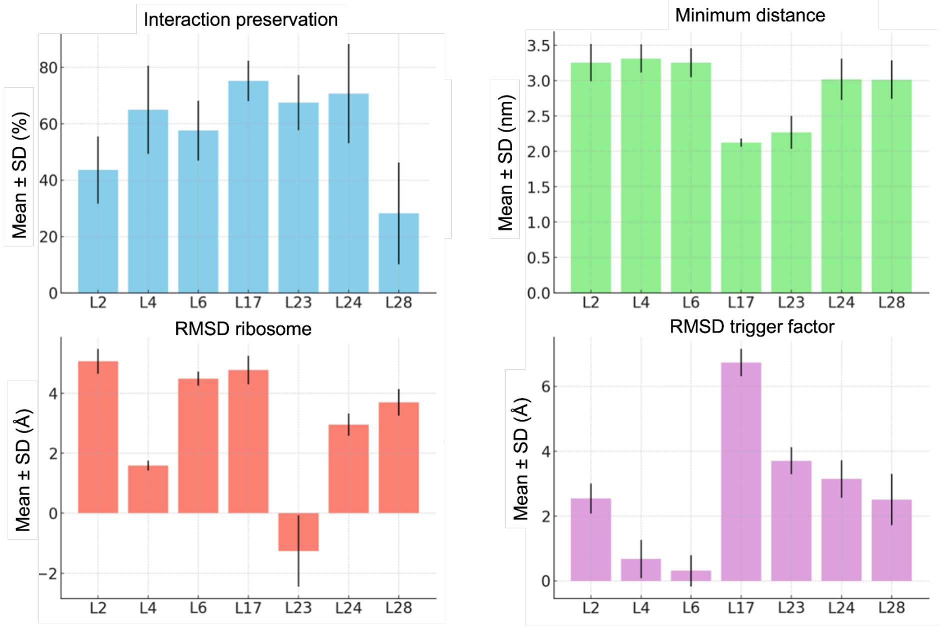

Figure S6. Screening of TF interactions with vestibule-adjacent ribosomal proteins based on MD simulations. (A) Left: minimum interatomic distances (Å) between each candidate ribosomal protein and the exit-tunnel vestibule, measured on the *E. coli* 70S ribosome (PDB: 7K00). uL23 (red) and uL24 (orange) directly line the vestibule (<2 nm). bL17 (cyan) is positioned at the peripheral rim (~3 nm), while uL2 (blue) lies at the base of the tunnel (~3.6 nm). uL28 (green), uL4 (pink), and uL6 (purple) are located on the solvent-exposed face, occupying more distal flanks (>5 nm). Right: Surface representation of the 70S ribosome (gray) with these seven screened proteins highlighted and labeled as mentioned before. **(B)** Quantitative metrics from 500 ns MD trajectories of docked TF–protein complexes: (i) interfacial contact retention (% of native contacts preserved), (ii) mean TF–protein centroid distance (Å), and (iii and iv) interface RMSD (Å). Analysis of the binding interfaces revealed that only the TF–uL23 and TF–bL17 pairs met our stability thresholds. Both exhibited a contact retention >60% and maintained an RMSD <6 Å, while also displaying minimum mean distances below 2.5 nm, identifying them as the most stable and structurally coherent binding partners for extended simulations.

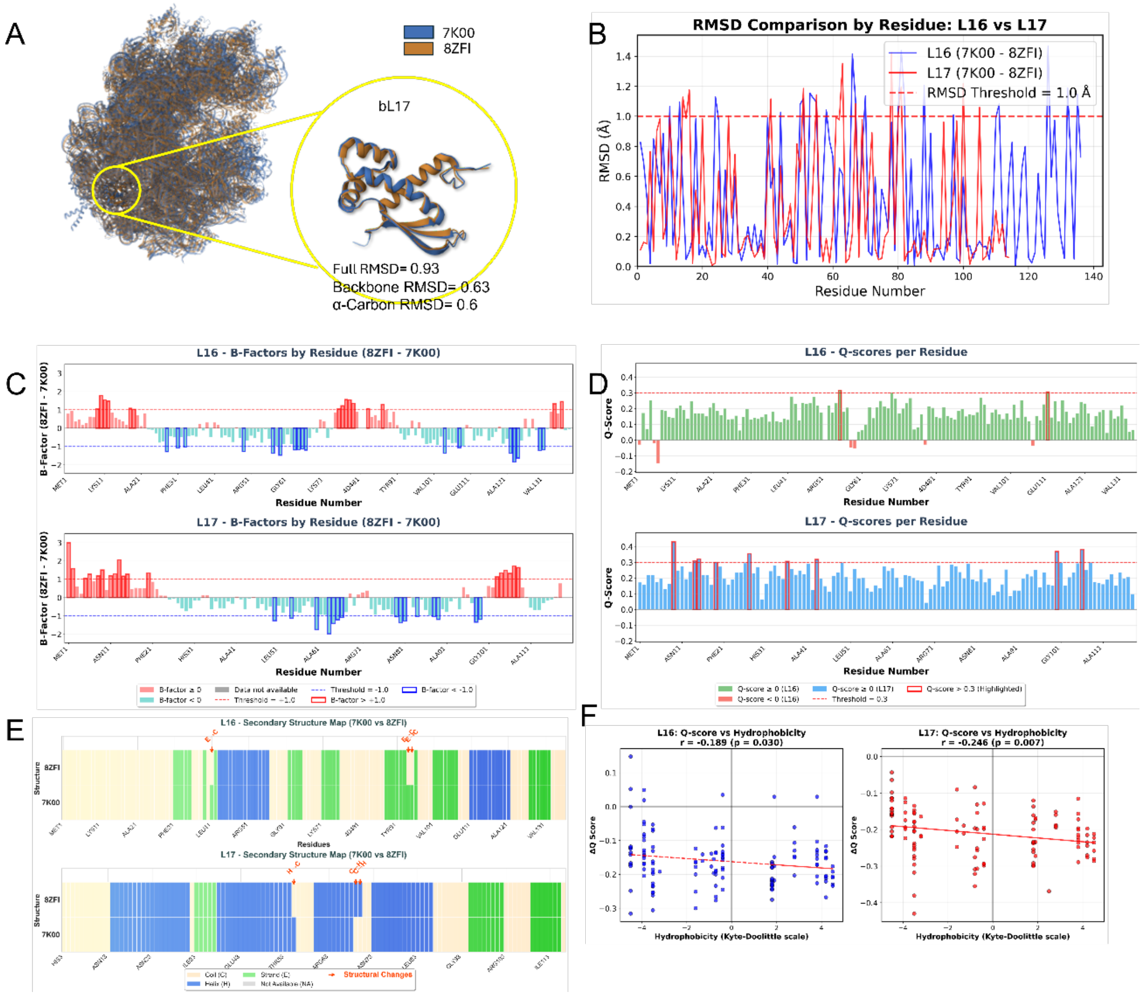

Figure S7. Comparative analysis of uL16 and bL17 between ribosome alone (PDB: 7K00) and TF/nascent chain–bound (PDB: 8ZFI) ribosome. (A) Structural alignment of 7K00 and 8ZFI, with a zoomed view highlighting bL17. **(B)** RMSD comparison by residue between 7K00 and 8ZFI for uL16 (blue line) and bL17 (red line). The RMSD threshold >1 Å is represented as a dashed red line. **(C)** Per-residue B-factor differences (8ZFI − 7K00) for uL16 and bL17, showing moderate increases in thermal motion in the bL17 central loop (residues 79–83) and C-terminal tail (residues 117–120), whereas uL16 shows minimal changes. **(D)** Per-residue ΔQ profiles (8ZFI − 7K00) for uL16 and bL17. A red dashed line at ± 1 indicates the threshold for significant B-factor deviations, and residues exceeding this threshold are highlighted. **(E)** Simplified secondary-structure comparison between 7K00 and 8ZFI, confirming overall conservation of secondary elements. **(F)** Scatter plot of ΔQ versus residue hydrophobicity (Kyte–Doolittle scale), indicating weak correlations (r ≈ −0.19 for uL16; r ≈ −0.25 for bL17). **(F)** B-factor differences mapped onto the ribosome structure. A red dashed line at ± 1 indicates the threshold for significant changes in residue flexibility, as B-factors report atomic mobility; residues exceeding this threshold are highlighted.

SUPPLEMENTAL VIDEO INFORMATION

Videos are available in both .avi and .tif formats on the Zenodo repository: https://doi.org/10.5281/zenodo.17185887

- **Video S1. 70S Ribosome – Orientation 1:** HS-AFM video of the *E. coli* 70S ribosome immobilized on a mica surface. Scan area: 150 × 150 nm^2 (300 × 300 pixels); scan speed: 1 s/frame. The video is displayed at 5× speed.
- **Video S2. 70S Ribosome – Orientation 2:** HS-AFM video of the *E. coli* 70S ribosome immobilized on a mica surface. Scan area: 100 × 100 nm^2 (300 × 300 pixels); scan speed: 1 s/frame. The video is displayed at 5× speed. Ps: Protein stalk.
- **Video S3. 70S Ribosome – Orientation 3:** HS-AFM video of the *E. coli* 70S ribosome immobilized on a mica surface. Scan area: 150 × 150 nm^2 (300 × 300 pixels); scan speed: 1 s/frame. The video is displayed at 5× speed. L1: L1 stalk; Ps: Protein stalk.
- **Video S4. 70S Ribosome – Orientation 4:** HS-AFM video of the *E. coli* 70S ribosome immobilized on a mica surface. Scan area: 100 × 100 nm^2 (300 × 300 pixels); scan rate: 1 frame/s. The video is displayed at 5× speed. CP: Central Protuberance; Ps: Protein stalk.
- **Video S5. 70S Ribosome – Orientation 5:** HS-AFM video of the *E. coli* 70S ribosome immobilized on a mica surface. Scan area: 150 × 150 nm^2 (300 × 300 pixels); scan rate: 1 frame/s. The video is displayed at 5× speed.
- **Video S6. TF MD Simulation – CHARMM36 + TIP3P (515 ns):** MD simulation of the TF monomer using the CHARMM36 force field with TIP3P water. The simulation was performed for 400 ns using GROMACS 2020. The TF structure (PDB: 1T11) was solvated in a cubic box with 0.15 M NaCl and periodic boundary conditions. After energy minimization, the system was equilibrated with 500 ps of NVT followed by 500 ps of NPT at 300 K using Langevin dynamics.
- **Video S7. TF MD Simulation – Amberff19SB + OPC (400 ns):** 400 ns MD simulation of TF monomer (PDB: 1T11), performed using the Amber ff19SB force field and OPC water model with the pmemd engine (Amber22). The system was simulated at 300 K with periodic boundary conditions and 0.15 M NaCl.
- **Video S8. TF monomer on a mica surface:** HS-AFM video of the TF monomer (5 nM) immobilized on a mica surface. Scan area: 100 × 100 nm^2 (300 × 300 pixels); scan speed: 1 s/frame. The video is displayed at 5× speed.
- **Video S9. TF dimer on a mica surface:** HS-AFM video of the TF dimer (5 μM) immobilized on a mica surface. Scan area: 100 × 100 nm^2 (300 × 300 pixels); scan speed: 1 s/frame. The video is displayed at 5× speed.
- **Video S10. 70S ribosome in the presence of TF:** HS-AFM video of the 70S ribosome after the addition of 1 μM TF on a mica surface. Scan area: 150 × 150 nm^2 (300 × 300 pixels); scan speed: 1 s/frame. The video is displayed at 5× speed.
- **Video S11. RNC-TF binding:** HS-AFM video of RNC complex after the addition of 1 μM TF on a mica surface. Scan area: 150 × 150 nm^2 (300 × 300 pixels); scan speed: 1 s/frame. The video is displayed at 5× speed. NC: Nascent Chain; TF: Trigger Factor.
- **Video S12. TF + Nascent Chain MD Simulation – CHARMM36 + TIP3P (1000 ns):** 1000 ns MD simulation of TF monomer (PDB: 1T11) interacting with the nascent chain, performed using NAMD 3.0.179 with the CHARMM36 force field and TIP3P water model. The system was simulated at 298 K and 1 atm pressure, applying periodic boundary conditions and Langevin dynamics.
- **Video S13. RNC-TF binding:** HS-AFM video of the RNC complex after the addition of 1 μM TF on a mica surface. Scan area: 150 × 150 nm^2 (300 × 300 pixels); scan speed: 1 s/frame. The video is displayed at 5× speed. TF: Trigger Factor.

- **Video S14. RNC-TF binding:** HS-AFM video of the RNC complex after the addition of 1 μM TF on a mica surface. Scan area: 200 × 200 nm^2 (300 × 300 pixels); scan speed: 1 s/frame. The video is displayed at 5× speed. TF: Trigger Factor.
- **Video S15. TF–bL17 MD Simulation – CHARMM36 + TIP3P (800 ns):** 800 ns MD simulation of the TF monomer (PDB: 1T11) docked to ribosomal protein bL17 (extracted from PDB: 7K00), following rigid-body docking with PIPER. Physically implausible docking poses were filtered prior to simulation. The system was modeled using the CHARMM36 force field and solvated in TIP3P water with 0.15 M NaCl. Simulations were run in GROMACS 2020 after energy minimization, NVT/NPT equilibration, and restraints on protein Cα atoms.
- **Video S16. TF–uL23 MD Simulation – CHARMM36 + TIP3P (800 ns):** 800 ns MD simulation of the TF monomer (PDB: 1T11) docked to ribosomal protein uL23 (extracted from PDB: 7K00), after filtering unrealistic poses generated via PIPER docking. Simulations were performed in GROMACS 2020 with the CHARMM36 force field, TIP3P water model, and 0.15 M NaCl. The system underwent energy minimization and NVT/NPT equilibration with Cα restraints.

DESCRIPTION OF DATASETS

- **Dataset S1.xlsx**: It contains raw and processed data corresponding to the measurements of ribosome dimensions (height, area, and volume) in different orientations, as shown in Figure 1B and Figure S1.
- **Dataset S2.xlsx**: It includes all quantitative analyses related to Figure 2, including: (A) time-dependent AR measurements of TF monomers, (B) histogram of TF conformational states, (C) SASA values from MD simulations (Amberff19SB and CHARMM36), (D) trajectory snapshots, and (E) boxplots comparing experimental and simulated aspect ratios.
    - Supplementary Figure 2: (A) height and area measurements of TF at two concentrations (5 nM and 5 µM), (B) time-dependent AR of TF dimers from HS-AFM recordings.
- **Dataset S3.xlsx:** Data supporting Figure 3: cross-sectional topography profiles and molecular contact analysis between TF and the nascent chain (MD simulations). Also includes:
    - Supplementary Figure 3B: dwell times for the first and second TF molecules bound to the RNC, with fitting parameters and residuals from exponential decay analysis.
    - Raw values and summary statistics for TF occupancy on ribosomes over time (Figure 4C).
    - Distance measurements and RMSD analyses for the TF–bL17 and TF–uL23 interfaces (Figures 4D–F).
    - Includes all-atom MD simulation data shown in Supplementary Figure 4, including histograms of minimum distances between TF (residues 300–392, 320, and 377) and the nascent chain, as well as hydrogen bond counts across replicates.
- **Dataset S4.xlsx:** It includes all quantitative metrics from Figure S6, from 500 ns MD trajectories of docked TF–protein complexes: (i) interfacial contact retention (% of native contacts preserved), (ii) mean TF–protein centroid distance (Å), and (iii) interface RMSD (Å).
- **Dataset S5.xlsx**. It contains all computational analyses supporting the structural comparison between ribosome alone (PDB: 7K00) and TF-bound ribosome (PDB: 8ZFI), including structural alignment and conservation, residue-specific conformational analysis, structural quality assessment, dynamic flexibility analysis, and secondary structure conservation.

REFERENCES FOR SI

(1) Saha, P.; Fernandez, I.; Sumbul, F.; Valotteau, C.; Kostrz, D.; Meola, A.; Baquero, E.; Sharma, A.; Portman, J. R.; Stransky, F.; Boudier, T.; Guardado-Calvo, P.; Gosse, C.; Strick, T.; Rey, F. A.; Rico, F. Modulation of SARS-CoV-2 Spike Binding to ACE2 through Conformational Selection. *Nat. Nanotechnol.* **2025**, *20*, 926–934.

(2) Schneider, C. A.; Rasband, W. S.; Eliceiri, K. W. NIH Image to ImageJ: 25 Years of Image Analysis. *Nat. Methods* **2012**, *9*, 671–675.

(3) Heath, G. R.; Kots, E.; Robertson, J. L.; Lansky, S.; Khelashvili, G.; Weinstein, H.; Scheuring, S. Localization Atomic Force Microscopy. *Nature* **2021**, *594*, 385–390.

(4) Heath, G. R.; Micklethwaite, E.; Storer, T. M. NanoLocz: Image Analysis Platform for AFM, High-Speed AFM, and Localization AFM. *Small Methods* **2024**, 2301766.

(5) Best, R. B.; Zhu, X.; Shim, J.; Lopes, P. E. M.; Mittal, J.; Feig, M.; MacKerell, A. D. Optimization of the Additive CHARMM All-Atom Protein Force Field Targeting Improved Sampling of the Backbone ϕ, ψ and Side-Chain χ_1 and χ_2 Dihedral Angles. *J. Chem. Theory Comput.* **2012**, *8*, 3257–3273.

(6) Jorgensen, W. L.; Chandrasekhar, J.; Madura, J. D.; Impey, R. W.; Klein, M. L. Comparison of Simple Potential Functions for Simulating Liquid Water. *J. Chem. Phys.* **1983**, *79*, 926–935.

(7) Tian, C.; Kasavajhala, K.; Belfon, K. A. A.; Raguette, L.; Huang, H.; Migues, A. N.; Bickel, J.; Wang, Y.; Pincay, J.; Wu, Q.; Simmerling, C. ff19SB: Amino-Acid-Specific Protein Backbone Parameters Trained against Quantum Mechanics Energy Surfaces in Solution. *J. Chem. Theory Comput.* **2020**, *16*, 528–552.

(8) Izadi, S.; Anandakrishnan, R.; Onufriev, A. V. Building Water Models: A Different Approach. *J. Phys. Chem. Lett.* **2014**, *5*, 3863–3871.

(9) Abraham, M. J.; Murtola, T.; Schulz, R.; Páll, S.; Smith, J. C.; Hess, B.; Lindahl, E. GROMACS: High Performance Molecular Simulations through Multi-Level Parallelism from Laptops to Supercomputers. *SoftwareX* **2015**, *1–2*, 19–25.

(10) Case, D. A.; Aktulga, H. M.; Belfon, K.; Cerutti, D. S.; Cisneros, G. A.; Cruzeiro, V. W. D.; Forouzesh, N.; Giese, T. J.; Götz, A. W.; Gohlke, H.; Izadi, S.; Kasavajhala, K.; Kaymak, M. C.; King, E.; Kurtzman, T.; Lee, T.-S.; Li, P.; Liu, J.; Luchko, T.; Luo, R.; Manathunga, M.; Machado, M. R.; Nguyen, H. M.; O'Hearn, K. A.; Onufriev, A. V.; Pan, F.; Pantano, S.; Qi, R.; Rahnamoun, A.; Risheh, A.; Schott-Verdugo, S.; Shajan, A.; Swails, J.; Wang, J.; Wei, H.; Wu, X.; Wu, Y.; Zhang, S.; Zhao, S.; Zhu, Q.; Cheatham, T. E.; Roe, D. R.; Roitberg, A.; Simmerling, C.; York, D. M.; Nagan, M. C.; Merz, K. M. AmberTools. *J. Chem. Inf. Model.* **2023**, *63*, 6183–6191.

(11) Darden, T. A.; Pedersen, L. G. Molecular Modeling: An Experimental Tool. *Environ. Health Perspect.* **1993**, *101*, 410–412.

(12) Humphrey, W.; Dalke, A.; Schulten, K. VMD: Visual Molecular Dynamics. *J. Mol. Graph.* **1996**, *14*, 33–38.

(13) Jumper, J.; Evans, R.; Pritzel, A.; Green, T.; Figurnov, M.; Ronneberger, O.; Tunyasuvunakool, K.; Bates, R.; Žídek, A.; Potapenko, A.; Bridgland, A.; Meyer, C.; Kohl, S. A. A.; Ballard, A. J.; Cowie, A.; Romera-Paredes, B.; Nikolov, S.; Jain, R.; Adler, J.; Back, T.; Petersen, S.; Reiman, D.; Clancy, E.; Zielinski, M.; Steinegger, M.; Pacholska, M.; Berghammer, T.; Bodenstein, S.; Silver, D.; Vinyals, O.; Senior, A. W.; Kavukcuoglu, K.; Kohli, P.; Hassabis, D. Highly Accurate Protein Structure Prediction with AlphaFold. *Nature* **2021**, *596*, 583–589.

(14) Phillips, J. C.; Braun, R.; Wang, W.; Gumbart, J.; Tajkhorshid, E.; Villa, E.; Chipot, C.; Skeel, R. D.; Kalé, L.; Schulten, K. Scalable Molecular Dynamics with NAMD. *J Comput. Chem.* **2005**, *26*, 1781–1802.

(15) Kozakov, D.; Brenke, R.; Comeau, S. R.; Vajda, S. PIPER: An FFT-based Protein Docking Program with Pairwise Potentials. *Proteins* **2006**, *65*, 392–406.

(16) Parrinello, M.; Rahman, A. Polymorphic Transitions in Single Crystals: A New Molecular Dynamics Method. *J. Appl. Phys.* **1981**, *52*, 7182–7190.

(17) Meng, E. C.; Goddard, T. D.; Pettersen, E. F.; Couch, G. S.; Pearson, Z. J.; Morris, J. H.; Ferrin, T. E. UCSF ChimeraX: Tools for Structure Building and Analysis. *Protein Sci.* **2023**, *32*, e4792.

(18) McGibbon, R. T.; Beauchamp, K. A.; Harrigan, M. P.; Klein, C.; Swails, J. M.; Hernández, C. X.; Schwantes, C. R.; Wang, L.-P.; Lane, T. J.; Pande, V. S. MDTraj: A Modern Open Library for the Analysis of Molecular Dynamics Trajectories. *Biophys. J.* **2015**, *109*, 1528–1532.

(19) Pintilie, G.; Zhang, K.; Su, Z.; Li, S.; Schmid, M. F.; Chiu, W. Measurement of Atom Resolvability in Cryo-EM Maps with Q-Scores. *Nat. Methods* **2020**, *17*, 328–334.
